# The incubation environment does not explain significant variation in heart rate plasticity among avian embryos

**DOI:** 10.1242/jeb.247120

**Published:** 2024-03-08

**Authors:** Alexandra G. Cones, Eve R. Schneider, David F. Westneat

**Affiliations:** Department of Biology, University of Kentucky, 101 Thomas Hunt Morgan Building, Lexington, KY 40506, USA

**Keywords:** Reaction norm, Thermal acclimation, Phenotypic plasticity, Birds, Growth

## Abstract

The conditions an organism experiences during development can modify how they plastically respond to short-term changes in their environment later in life. This can be adaptive because the optimal average trait value and the optimal plastic change in trait value in response to the environment may differ across different environments. For example, early developmental temperatures can adaptively modify how reptiles, fish and invertebrates metabolically respond to temperature. However, whether individuals within populations respond differently (a prerequisite to adaptive evolution), and whether this occurs in birds, which are only ectothermic for part of their life cycle, is not known. We experimentally tested these possibilities by artificially incubating the embryos of Pekin ducks (*Anas platyrhynchos domesticus*) at constant or variable temperatures. We measured their consequent heart rate reaction norms to short-term changes in egg temperature and tracked their growth. Contrary to expectations, the early thermal environment did not modify heart rate reaction norms, but regardless, these reaction norms differed among individuals. Embryos with higher average heart rates were smaller upon hatching, but heart rate reaction norms did not predict subsequent growth. Our data also suggests that the thermal environment may affect both the variance in heart rate reaction norms and their covariance with growth. Thus, individual avian embryos can vary in their plasticity to temperature, and in contrast to fully ectothermic taxa, the early thermal environment does not explain this variance. Because among-individual variation is one precondition to adaptive evolution, the factors that do contribute to such variability may be important.

## Teaser

**Summary:** Individual avian embryos exhibit significant variation in how they respond to temperature, which cannot be explained by the incubation they experienced.

## INTRODUCTION

All organisms exhibit reversible plasticity: temporary phenotypic responses to short-term changes in the environment that occur within their lifetime (Snell-Rood, 2013). Often, different populations of the same species have evolved divergent levels of reversible plasticity, with organisms from some populations responding more strongly and organisms from other populations responding less strongly to variation in their environment. These differences may be adaptive. For example, high predation rates favor a faster pace-of-life for Trinidadian guppies (*Poecilia reticulata*), and indeed, guppy populations with higher predation rates have evolved higher plasticity of their litter size in response to food, which allows them to accelerate their pace-of-life when possible (Gordon et al., 2017). Although it is obvious that populations often experience different environments, environmental variation often exists within populations, so individuals might be expected to ‘acclimate’ to their specific developmental environment by adjusting their reversible plasticity in response (a form of multidimensional plasticity; Westneat et al., 2019). Indeed, it is well known that the developmental environment can modify later reversible plasticity across taxa (Beaman et al., 2016). Nevertheless, we do not fully understand the environments that lead to the evolution of the mechanisms that allow for this reversible plasticity acclimation, nor which traits are more likely to exhibit this ability.

Temperature, a dimension of the environment that varies highly over time and space, constrains the ability of organisms to acquire and utilize energy for survival and reproduction. For ectotherms, this effect is clear: a change in body temperature elicits a reversible plastic change in physiological processes such as metabolic rate, heart rate and growth. These plastic responses can be described quantitatively by constructing a regression of the trait across a gradient for a specific dimension of the environment and assessing the intercept and slope of this regression (a ‘reaction norm’; Woltereck, 1909; Nussey et al., 2007). An organism's expected phenotype in the ‘average’ environment is the intercept of this function, and the slope of their phenotype over the environmental gradient is the magnitude of plasticity. Using this reaction norm approach, we have previously demonstrated that individuals within endothermic populations can vary in both the intercept and slope of their reversible responses to temperature during ectothermic life stages: house sparrow embryos (*Passer domesticus*) varied in both their average heart rate and their heart rate plasticity to egg temperature, and further, these reaction norms may be modified by the developmental environment (Cones and Westneat, 2023). Work in fully ectothermic species has demonstrated that the early thermal environment can modify later, reversible metabolic responses to temperature (Fangue et al., 2009; Schaefer and Walters, 2010; Seebacher and Grigaltchik, 2015; Sun et al., 2015; Campbell-Staton et al., 2018), and that this flexibility may be adaptive (Terblanche et al., 2009). However, whether this thermal acclimation of reversible plasticity has evolved in birds, which are ectothermic for part of their life cycle but become endothermic and have different ecological pressures and life histories, is not well studied. Because selection pressures at the embryonic and adult life stages may conflict, investigating whether, when and how this acclimation of reversible plasticity occurs in avian species will improve our understanding of evolution of phenotypic plasticity more generally.

Temperature during embryonic development has strong effects on avian growth and survival through its effects on metabolic output, activity and growth (DuRant et al., 2013). Cold incubation temperatures prolong embryonic development, increase predation risk and can result in poorer quality post-hatching (Conway and Martin, 2000; Angilletta et al., 2004; Olson et al., 2006; Jonsson et al., 2022). Consequently, it may benefit avian embryos in cold conditions to accelerate development, thus increasing their overall metabolic output and increasing their metabolic plasticity to short-term changes in temperature, in order to take advantage of intermittent warm conditions (Terblanche et al., 2009). Because accelerating development seems to incur downstream costs (e.g. through reduced longevity; Ricklefs et al., 2017), embryos should only do this when incubation conditions are sufficiently poor. Because incubation conditions (e.g. egg temperatures) vary within avian populations (e.g. Cones et al., 2021), we predict that avian embryos should have evolved the ability to acclimate their reversible plasticity to temperature based on the thermal environment they experience early in development.

To test whether avian embryos do adjust reversible metabolic plasticity to temperature as a function of their incubation environment and whether they vary in this plasticity, we manipulated the early thermal environment of Pekin duck (*Anas platyrhyncos domesticus*) embryos and measured their consequent metabolic plasticity to temperature and their growth. We used Pekin ducks because: (1) large numbers of eggs can be sourced at the same time at the same developmental window, (2) embryos develop reliably in artificial incubators, and (3) many individuals can be raised post-hatching in a common garden environment. Specifically, we artificially incubated duck embryos in either a constant warm or a variable cooling thermal regime, quantified their heart rate reaction norms to temperature, and analyzed the covariance between their heart rate reaction norms and growth. Although heart rate might not always be a reliable proxy of metabolic rate per se (Butler et al., 2004; Clark et al., 2006; Green, 2011; Sartori et al., 2017), it is an important factor regulating the conversion of resources to energy and tissues as well as the speed of development (Du and Shine, 2015). Consequently, we predicted that (1) average heart rate and heart rate plasticity to temperature would vary among duck embryos, and (2) embryos in the variable cooling regime would develop higher heart rates and greater heart rate plasticity to temperature than those in the constant warm regime because (3) embryos with higher heart rates and greater heart rate plasticity to temperature would develop faster both pre- and post-hatching. During data analysis, we noticed unique patterns of variance in heart rate reaction norms between the two experimental regimes. Consequently, we investigated possible effects of the thermal environment during incubation on the variance and covariance of heart rate reaction norms and growth.

## MATERIALS AND METHODS

### Egg sourcing and preparation

Pekin duck (*Anas platyrhyncos domesticus* Linnaeus 1758) eggs were sourced from Maple Leaf Farms (located in Cromwell, IA, USA). Eighty eggs were shipped overnight in two batches on different dates from various, unknown mothers. Prior experience indicated that minimal development occurs during shipping. Once the eggs arrived, they were washed with deionized water and allowed to air dry. For each batch, A.G.C. numbered each egg with a unique ID with a fine-tipped Sharpie (*N*=24 eggs for each batch (*N*=2), but this sample was reduced for the full experiment, see below). Before the onset of artificial incubation, A.G.C. took measures of egg mass using a digital scale (to the nearest 0.01 gram) and egg volume (length×width^2^, mm) using calipers.

All procedures were consistent with approved University of Kentucky Insitutional Animal Care and Use Committee protocol 2021-3810.

### Artificial incubation regimes

We arbitrarily assigned individual eggs to one of two artificial incubation regimes: constant warm incubation and variable cooling incubation. In batch 1, incubator 1 was the warm regime and incubator 2 was the cooling regime. For batch 2, these regimes were swapped between the incubators. The incubators (Brinsea Ovation 28 Advance digital egg incubators) were placed side-by-side near a window to allow natural light cycles. Both were set to 37.5°C, 70% humidity and turning every other hour. We set the cooling regime to intersperse these optimal incubation conditions with bouts of cooling during daylight hours. We plugged the cooling incubator into a manual outlet timer that turned off the incubator every other hour during daylight hours (06:00–18:00 h) for 45 min each to cool eggs and simulate incubation recesses (temperatures dropped from 37.5°C to ∼30°C, as measured by a thermal probe in the incubator). Checks confirmed that this cooling did not cause eggs to reach physiological zero (Webb, 1987). On day 7 of incubation, when embryonic development becomes discernable by eye, eggs were candled, leading to the removal of one egg in each batch that did not show any signs of development. To not exceed target sample sizes imposed by time limitations, we also arbitrarily removed and euthanized five eggs in batch 1, and one egg in batch 2, leaving 18 viable eggs in batch 1 and 22 in batch 2, which were split equally between incubation treatments.

### Heart rate and egg mass measures

In order to assess embryonic responses to cooling, we measured instantaneous embryonic heart rate, which can be assayed reliably using a digital heart rate monitor (Pollard et al., 2016). We took repeated measures of embryonic heart rate and concurrent egg temperature during short-term cooling bouts. Specifically, once embryonic heart rate was measurable (incubation day 13), We individually removed each egg from the incubator (when it was at 37.5°C; i.e. cooling treatment eggs were not removed during the cooling phase) and placed it into an Egg Buddy digital heart rate monitor (Avitronics MK2). The monitor uses infrared light to non-invasively report continuous embryonic heart rate in beats per minute. Once the egg was in the monitor, we started a timer and recorded the starting egg temperature using a handheld infrared thermometer (FLIR TG167). Upon removal from the incubator, eggs would continuously drop in temperature until their return to the incubator. Every subsequent 3 min, we recorded a heart rate measurement along with a concurrent measurement of egg temperature. This procedure took approximately 30 min per egg (*N*=10 measures). If the monitor did not display a reliable heart rate at the scheduled time, we recorded heart rate as soon as possible thereafter, and if we could record no reliable heart rate measurement before the next time interval, we entered an ‘NA’ for that time interval (*N*=259 instances out of 880 total measures). We took measurements until either 30 min had passed or until the egg cooled to ∼30°C. After we took the last measurement, we immediately returned the egg to its incubator. Each egg was cooled and measured in this way twice during incubation (age range=incubation days 13–18). These measures were taken around the mid-point of embryonic development, and thus we do not expect our measures to be biased by production of heat by the embryos themselves. We could not measure all eggs within a batch in a single day, so eggs within a batch were divided into three equal ‘blocks’ (batch 1: *N*=6 eggs per block; batch 2: *N*=8 eggs; batch 3: *N*=8 eggs). Each block comprised an equal number of eggs from each treatment. We measured heart rates on incubation days 13 and 16 for block 1, 14 and 17 for block 2, and 15 and 18 for block 3. We measured eggs from each block at the same time of day on subsequent days. We also measured the masses of each egg on incubation days 0, 6, 18, 21 and 24 (with additional measures every day after day 24 until hatching, maximum age at hatching=29 days).

### Measuring incubation duration and preparation for hatching

Once any eggs within an incubator showed signs of external pipping, we moved the whole incubator group to the brooder for hatching. Before this move, we weighed the eggs for a final time and internally dyed them to allow for individual identification post-hatching. To do so, we candled to determine the location of the embryo and injected the egg with 0.2 ml of Fast Green dye (2% in ddH­_2_O) with a 22-gauge needle while avoiding puncturing the embryo. After injection, we covered the hole with a small square of surgical tape (∼2 mm^2^). Finally, we moved the eggs into the brooder, in a segmented tray, which was set 37.5°C and 70% humidity. We checked the eggs in the brooder roughly every 4 h between 08:00 and 20:00 h for signs of hatching. The incubation duration of each individual was quantified as the incubation age at which they hatched (range=26.7–29.35 days).

### Measuring post-hatching growth

After an egg hatched, we measured duckling (left) tarsus length to the nearest 0.1 mm using calipers. We individually marked each duckling with a uniquely colored zip tie on each leg. We checked these color bands daily and they were routinely replaced as needed (∼every 3 days) to ensure that bands did not become tight as the ducklings grew. Once ducklings were large enough, we gave them permanent plastic color bands. Ducklings were kept in the brooder no longer than 24 h. We removed groups of hatched ducklings in that 24 h window from the brooder, placed them into a covered animal carrier, and immediately transported them to the aviary facilities (University of Kentucky Ecological Research and Education facility) in a climate-controlled vehicle (∼15 min). Upon arrival, newly hatched ducklings were raised in large clear bins in a climate-controlled room (70°C, 25% humidity). The bins contained chopped straw (replaced twice daily), a Brinsea brooder (Brinsea Safety Eco 600), continuously accessible waterers, Mazuri duck starter diet (supplemented with powdered Niacin) and duckling grit. Once ducklings were capable of feeding and moving around, we moved the ducklings to outdoor enclosed aviaries. Each group of ducklings spent an equivalent number of days in the climate-controlled room before moving outside. Outdoor aviaries also contained straw bedding, brooders, grit, and ad libitum access to water and duck feed. We took daily measures of duckling tarsus length during this post-hatching growth period.

### Statistical analyses

We estimated the variance of embryonic heart rate reaction norms, the effect of incubation regime on these traits, and the covariance between these heart rate reaction norms and pre- and post-hatching growth traits. We obtained 7–20 measures of heart rate and 5–7 measures of egg mass per egg during incubation. Thirty-one eggs hatched (constant: *N*=18 eggs; cooling: *N*=13 eggs). We also obtained daily measures of duckling tarsus up to 9–30 days post-hatching (ducklings at the low end of this range were removed by extrinsic causes: predation event, *N*=6; quarantined owing to suspected infection, *N*=1). Although we obtained measures of duckling tarsus up to day 57 post-hatching, we subset the tarsus data to days 0–30 because growth remained linear during that period. Using these repeated measures, we analyzed the effect of incubation regime on embryonic heart rate reaction norms, the association between these reaction norms and growth, and the effect of regime on variance in embryonic heart rate reaction norms and their covariance with growth using a series of Bayesian linear mixed-effect models using the MCMCglmm package (Hadfield, 2010) in R 4.3.1 (https://www.r-project.org/). All models were run with weakly informative inverse-gamma priors (prior degree of belief=0.002) for random effects (Lemoine, 2019) and for 100,000 iterations with a burn-in of 1000 iterations and a thinning interval of 10, with one chain. Typically, parameter estimates from Bayesian models are considered important if the 95% credible intervals do not overlap zero, but random effect variance estimates cannot be negative, thereby ensuring that credible intervals do not overlap zero (Pick et al., 2023). Instead, we assessed whether the median and mode were similar compared with the breadth of the distribution. We also examined the posterior distributions visually and used the combination of information to assess whether the posterior distribution was symmetrical and did not contain an excess of near-zero values (see Supplementary Materials and Methods and Figs S1–S3 for code and plots of posterior distributions). All figures were made in ggplot2 (Wickham, 2016).

### Sources of variance in embryonic heart rate reaction norms

To estimate the among-individual variance in average embryonic heart rate and heart rate plasticity to temperature, we fitted a model with a fixed effect of egg temperature (eggtemp), which was measured over multiple instances (subscript *i*) for each egg (subscript *j*) (Eqn 1)*. *We denoted effect intercepts with subscript 0, and effect slopes with subscript 1. We fitted a random intercept for egg ID (eggID), which estimates the variance in average heart rate among individuals, and we fitted a random slope term of egg temperature by egg ID, which estimates variance in the plasticity of heart rate to egg temperature among individuals (Eqn 1). To estimate intercepts of heart rate at the average egg temperature as opposed to at an egg temperature of 0°C, we centered egg temperature to the population mean. We also included incubation day (incday) as a fixed covariate in this model as we expected heart rate to change with embryonic age. To test the effect of incubation treatment (incregime) on the plasticity of heart rate in response to egg temperature (eggtemp) we included their interaction in the model as well (Eqn 1):
(1)

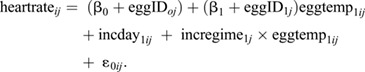


### Sources of variance in growth

To test the effects of incubation regime on growth, we ran three separate linear models (one for incubation duration, one for egg mass and one for duckling tarsus). The model of incubation duration included a fixed effect of incubation regime (incregime) (Eqn 2):
(2)

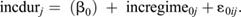


The model of egg mass included a fixed effect of incubation day (incday), incubation regime (incregime), their interaction, a random intercept at the among-egg level (eggID) (which estimates individual deviance from the sample mean in initial egg mass) and a random slope at the egg level (eggID) with respect to incubation day (incday) (which estimates each individual's rate of egg mass loss relative to the population mean egg mass loss over the incubation period) (Eqn 3). Variance in both random effects was estimated along with their credible intervals. The analysis equation was thus:
(3)

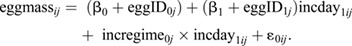


The model of duckling tarsus also included a fixed effect of duckling age (ducklingage), incubation regime (incregime), their interaction, a random intercept at the individual level (eggID), producing estimates for each individual and the variance in hatching size, and a random slope at the individual level (eggID) with respect to duckling age (ducklingage), which estimates each individual's rate of structural growth post-hatching and the among-individual variance in growth (Eqn 4): 
(4)

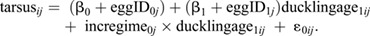
We analyzed duckling growth as a linear function, restricted to the first 30 days post-hatch, because we found little evidence of non-linearity during this period and it allowed uncertainty in the estimates of growth to remain in the statistical analyses.

### Links between heart rate reaction norms and growth

In order to estimate the correlation between heart rate reaction norms and growth at the among-individual level, we ran a series of targeted bivariate models. We used multiple bivariate models as opposed to a single multivariate model as we wanted to estimate only the specific correlations between heart rate reaction norms and each measure of growth. For each bivariate comparison, we specifically estimated the correlations between heart rate reaction norms (average heart rate and heart rate plasticity to temperature) and each growth measure (incubation duration, egg mass reaction norms and duckling tarsus reaction norms). To do so, we ran a bivariate comparison of Eqns 1 and 2, Eqns 1 and 3, and Eqns 1 and 4. Because we were interested in the general relationships between heart rate reaction norms and growth, and because incubation regime did not affect heart rate reaction norms, we removed the incubation regime fixed effect from Eqns 1–4. Additionally, to estimate the relationships between these traits at the among-individual level, we needed to add a random intercept term (eggID) to Eqn 2 for the bivariate analysis. To facilitate comparisons among variables with different measurement scales, we *z*-standardized heart rate, egg temperature, incubation duration, egg mass and tarsus length. Correlation estimates were deemed potentially important if the 95% credible intervals did not overlap zero.

### Effects of incubation regime on the (co)variance of heart rate reaction norms and growth

To test the effect of incubation regime on the variance in heart rate reaction norms among embryos, we ran the heart rate model (Eqn 1) for each experimental group (constant warm versus variable cooling) separately [and thus, removed the treatment fixed effect (incregime) from the model], and then compared the credible intervals of the random terms. The estimates of variance and covariance from the two incubation regimes were deemed to be potentially different if the median (co)variance estimate of one incubation regime did not fall within the 95% credible interval of the other incubation regime. Similarly, we tested the effect of incubation regime on the covariance of heart rate and growth by running the bivariate models outlined above using data from the warm incubation regime and then the cooling incubation regime separately, and comparing the resulting credible intervals for correlation estimates.

## RESULTS

### Sources of variance in embryonic heart rate reaction norms

**Fig. 1.**
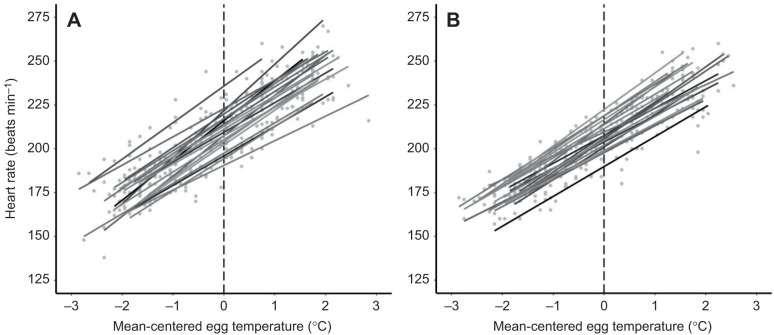
**Among-individual variance in heart rate reaction norms in two incubation regimes.** Both panels show the positive effect of population mean-centered egg temperature on embryonic heart rate. In both panels, each regression line represents the heart rate reaction norm of an individual embryo and the dashed line represents the location of the heart rate intercept. Data from the constant warm incubation regime are drawn in A, whereas data from the variable cooling incubation regime are drawn in B.

As expected, embryonic heart rate increased with increasing egg temperature (Table 1, Fig. 1). Contrary to our predictions, we found no difference in average heart rate or heart rate plasticity to temperature between the two incubation regimes (Table 1). Nevertheless, average heart rate varied significantly among individual embryos (Table 1, Fig. 1). Further, individual embryos varied in their heart rate plasticity to egg temperature (Table 1, Fig. 1). Despite this variance in both average heart rate and heart rate plasticity to temperature at the among-individual level, we found no evidence of covariance between an individual's average heart rate and their heart rate plasticity to temperature (Table 1, Fig. 1). Heart rate also varied between measurements within individuals, and this variance remains unexplained by incubation regime, egg temperature, incubation day or egg identity (residual variance; Table 1).

**Table 1.**
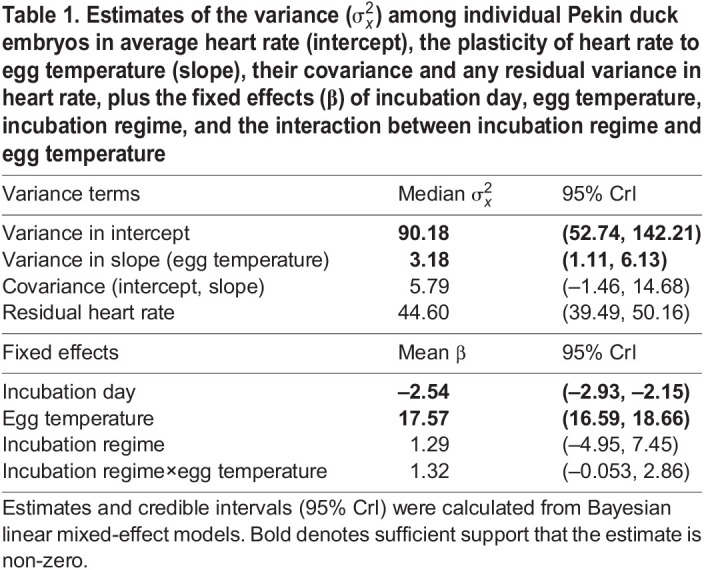
**Estimates of the variance (** 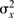 **) among individual Pekin duck embryos in average heart rate (intercept), the plasticity of heart rate to egg temperature (slope), their covariance and any residual variance in heart rate, plus the fixed effects (β) of incubation day, egg temperature, incubation regime, and the interaction between incubation regime and egg temperature**

### Sources of variance in growth

**Fig. 2.**
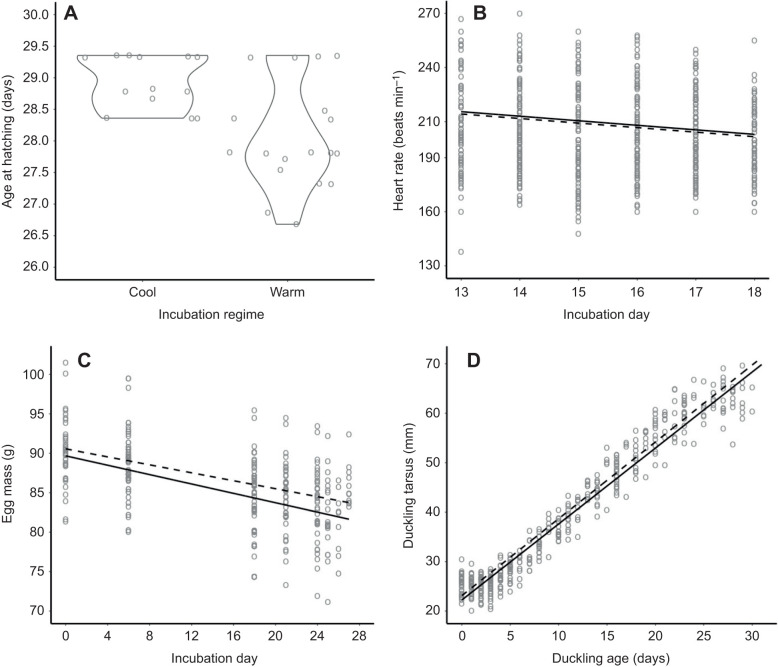
**Effects of incubation regime on embryo and duckling growth.** (A) Individuals in the variable cooling incubation regime took longer to hatch on average compared to those in the constant warm incubation regime. In panels B through D, the solid regression line represents the average response in the warm incubation regime whereas the dashed line represents the average response in the cooling incubation regime. (B) Embryonic heart rate reduced between incubation days 13 to 18, but this pattern did not differ between the incubation regimes. (C) Egg mass declined over the entire incubation period, but this effect was stronger in the warm incubation regime. (D) Duckling tarsus increased steadily post-hatching, but incubation regime did not modify this post-hatching growth trajectory.

Although incubation regime did not affect heart rate reaction norms, it did affect embryonic growth. Unsurprisingly, embryos in the constant warm incubation regime took less time to hatch than those in the variable cooling regime (Table 2, Fig. 2A). In the latter half of the incubation period (when heart rates were measured), incubation regime did not alter the reduction in heart rate with increasing incubation day (Table 2, Fig. 2B). Eggs in the variable cooling regime lost less egg mass over the incubation period compared with those from the constant warm incubation regime (Table 2, Fig. 2C). Post-hatching, incubation regime did not affect growth rates (Table 2, Fig. 2D).

**Table 2.**
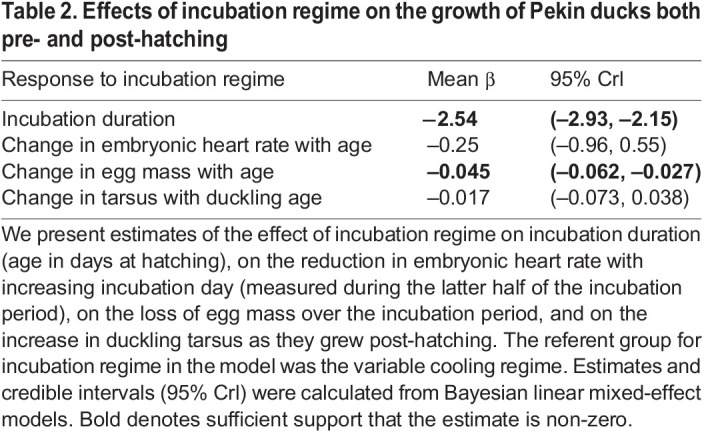
Effects of incubation regime on the growth of Pekin ducks both pre- and post-hatching

### Links between heart rate reaction norms and growth

Contrary to expectations, embryonic heart rate did not predict incubation duration (Table 3). Nevertheless, embryos with higher average heart rates lost more egg mass over the incubation period, regardless of their starting egg mass (Table 3). Further, ducklings that had higher average heart rates as embryos were smaller upon hatching but did not differ in their growth rates compared with their peers (Table 3). The plasticity of embryonic heart rate to temperature did not correlate with any of our measures of growth (Table 3).

**Table 3.**
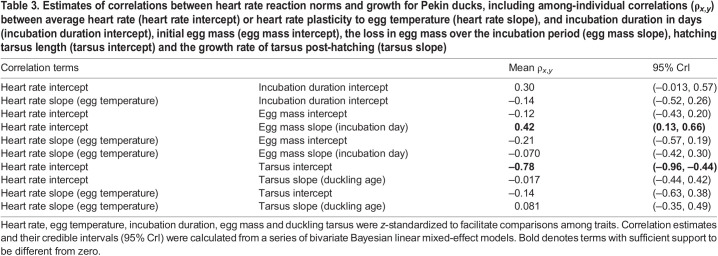
Estimates of correlations between heart rate reaction norms and growth for Pekin ducks, including among-individual correlations (ρ*_x_*_,*y*_) between average heart rate (heart rate intercept) or heart rate plasticity to egg temperature (heart rate slope), and incubation duration in days (incubation duration intercept), initial egg mass (egg mass intercept), the loss in egg mass over the incubation period (egg mass slope), hatching tarsus length (tarsus intercept) and the growth rate of tarsus post-hatching (tarsus slope)

### Effects of incubation regime on (co)variance of heart rate reaction norms and growth

Although average heart rate varied among embryos, we found no evidence that variance in heart rate among individuals differed between the two incubation regimes (Table 4). In contrast, the variance in heart rate plasticity to temperature among embryos was higher in the warm incubation regime compared with the cooling incubation regime (the median variance in the constant warm regime was outside the credible interval of the variable cooling regime; Table 4, Fig. 1). The negative relationship between average heart rate and hatching size was stronger in the constant warm regime (Table 4). In contrast, the correlation between heart rate plasticity and hatching size seemed to go in opposite directions in the two incubation regimes: embryos in the constant warm incubation regime with higher heart rate plasticity tended to hatch smaller, whereas those in the variable cooling incubation regime with higher heart rate plasticity tended to hatch larger (Table 4). Although these relationships were significantly different from each other (the correlation estimates of one regime were not encapsulated in the credible intervals of the other regime), they were not different from zero.

**Table 4.**
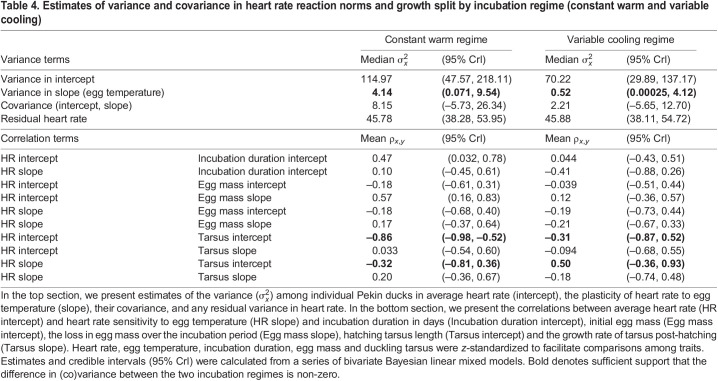
Estimates of variance and covariance in heart rate reaction norms and growth split by incubation regime (constant warm and variable cooling)

## DISCUSSION

Our experiment revealed that individual duck embryos differed in their heart rate reaction norms to temperature, and further, that early thermal conditions were not a detectable source of this variance. Furthermore, the variation in average heart rates or heart rate plasticity did not covary significantly with most of our measures of growth. One exception was that embryos with higher average heart rates lost more mass over the incubation period and hatched at a smaller size. We also observed that incubation regime had some influence on the among-individual variance in heart rate reaction norms and their covariance with growth: both the variance in heart rate plasticity and the relationship between heart rate reaction norms and hatching size differed between the two incubation regimes. Overall, these results confound our expectation, based on work in fully ectothermic taxa, that thermal acclimation would drive variation in embryonic plasticity, and so raise some new questions about when we expect the acclimation of reversible plasticity to evolve.

Our finding that average heart rates varied among embryos adds to the small body of work that has found embryonic heart rate variance within wild populations (Burggren et al., 1994, 2003; Vedder et al., 2017; Sheldon et al., 2018; Cones and Westneat, 2023). Further, we found significant variance among embryos in their plasticity to temperature. Variance in metabolic plasticity to temperature has been documented in other taxa (Lighton et al., 2001; Nespolo et al., 2003; Terblanche et al., 2007; Kar et al., 2021), and indirect evidence from avian species suggests that they vary in their plasticity to temperature as well (via differences in growth when controlling for temperature and mass) (Martin et al., 2015). We previously assessed variance in heart rate reaction norms in free-living house sparrows (*Passer domesticus*) and found that variation in maternal age was associated with variation in embryonic heart rate plasticity to temperature (Cones and Westneat, 2023), suggesting that the developmental environment might be altering embryonic heart rate plasticity. We expected the early thermal environment to be a likely source of variance in avian embryonic heart rate plasticity to temperature, but in the present study, we found that embryonic ducks did not acclimate their heart rate plasticity in response to their incubation environment. However, we cannot rule out the possibility that a more extreme difference in thermal regimes may have induced thermal acclimation. Regardless, we found significant variance that cannot be explained by the thermal treatments.

Genes are a possible source of the observed variance in heart rate reaction norms, as seen in some fully ectothermic species (e.g. Fangue et al., 2009; Terblanche et al., 2009; Díaz et al., 2012; Hall and Warner, 2019). However, parental effects, for example via manipulation of embryonic hormones, can also produce significant variance in offspring metabolism/growth, and much of the evidence for this comes from birds (Groothuis et al., 2005). Indeed, the heart rate plasticity of house sparrow (*Passer domesticus*) embryos was positively related to the age of their mothers (Cones and Westneat, 2023), supporting the parental effects hypothesis. Yet, in the same study, maternal age did not explain significant variation in average embryonic heart rate, and in both that study and this experiment, average embryonic heart rate did not covary with heart rate plasticity to temperature. Because these two heart rate traits appear to vary independently, their causes of variance may differ. To untangle the sources of variance in multiple metabolic traits, and furthermore, to assess their fitness consequences, we need more studies that directly quantify both average heart rate and heart rate plasticity to the environment, among other measures of metabolic output.

Contrary to expectations, we found that duck embryos with higher embryonic heart rates did not develop faster, they lost more mass over the incubation period, and they were structurally smaller upon hatching. Previous work demonstrates that the relationship between temperature, heart rate, metabolism and development in oviparous species can vary (Zuo et al., 2012). For example, lower temperatures delay development, which can lead to larger size upon hatching (known as the ‘temperature–size rule’; Atkinson, 1994). In contrast, cold temperatures can force embryos to waste the finite resources within the egg on protracted development, necessitating more total heart beats before hatching and, consequently, smaller size upon hatching (known as the ‘reverse temperature–size rule’), which has been observed in both avian and non-avian reptiles (Du et al., 2009). Our results suggest that these ideas do not capture the full complexity of the relationships between the thermal environment and the growth of ectothermic embryos. Although duck embryos in the constant warm incubation regime developed faster, those in the warm regime did not have higher heart rates, and we found that increased heart rate did not accelerate development. In contrast, in zebra finches (*Taeniopygia guttata*), embryonic heart rate is positively associated with accelerated growth both pre- and post-hatching (Vedder et al., 2017; Sheldon and Griffith, 2018). The relationship between heart rate, metabolic rate and growth rate is thus not straightforward. For example, egg temperatures can modify the relationship between metabolic rate and growth rate in avian embryos (Martin et al., 2013; Glazier, 2015b; Schulte, 2015). However, we did not find evidence that the relationship between heart rate and incubation duration differed between the two incubation regimes (Table 4). Further, traits such as heart mass can decouple heart rate from metabolic output (Du et al., 2010), and experimental manipulations can modify (resting) metabolic rate without modifying growth rates (Tobler et al., 2007). Nevertheless, our results suggest that variance in heart rate is still functionally meaningful: embryonic heart rate was associated with hatching quality, and may have other, unmeasured consequences for phenotype. Further, incubation conditions seem to have had some effect on the relationship between heart rate and growth trajectories. These results highlight the need for more work untangling the complex relationships between the environment, heart rate, metabolic rate and growth.

That average heart rate and heart rate plasticity varied among individuals and is likely correlated with life-history and reproductive traits (based on a large body of previous work: Ricklefs and Wikelski, 2002; White and Seymour, 2004; Biro and Stamps, 2010; Glazier, 2015a,b; White et al., 2022) suggests that the optimal metabolic output is likely context dependent (Burton et al., 2011). Although we found that the thermal environment did not alter embryonic heart rate reaction norms, it did alter the variance in heart rate plasticity, and the relationship between heart rate reaction norms and hatching size. Whether the thermal environment affects the relationship between embryonic heart rate and other functional traits, and their variance, is not clear but is important for understanding the evolution of these traits. These kinds of effects could modify the responses of populations to climate change; thermal effects could alter the variation in phenotypic responses to the environment and the relationship between those traits and fitness, changing the selection on individual traits in quickly changing environments. These results suggest that the environmental effects on metabolism and growth are complex, but we regard them as important for understanding the evolution of populations in variable environments. A multivariate analytical framework coupled with experimental manipulations in taxa with different life histories may offer a good opportunity to further disentangle these nuances.

## Supplementary Material

10.1242/jexbio.247120_sup1 Supplementary information

Table S1. Raw data.

## References

[JEB247120C1] Angilletta, M. J., Steury, T. D. and Sears, M. W. (2004). Temperature, growth rate, and body size in ectotherms: fitting pieces of a life-history puzzle. *Integr. Comp. Biol.* 44, 498-509. 10.1093/icb/44.6.498 21676736

[JEB247120C2] Atkinson, D. (1994). Temperature and organism size: a biological law for ectotherms. *Adv. Ecol. Res* 25, 1-58. 10.1016/S0065-2504(08)60212-3

[JEB247120C3] Beaman, J. E., White, C. R. and Seebacher, F. (2016). Evolution of plasticity: mechanistic link between development and reversible acclimation. *Tree* 31, 237-249. 10.1016/j.tree.2016.01.004 26846962

[JEB247120C4] Biro, P. A. and Stamps, J. A. (2010). Do consistent individual differences in metabolic rate promote consistent individual differences in behavior? *Tree* 25, 653-659. 10.1016/j.tree.2010.08.003 20832898

[JEB247120C5] Burggren, W. W., Tazawa, H. and Thompson, D. and Thompson, D. (1994). Genetic and maternal environmental influences on embryonic physiology: intraspecific variability in avian embryonic heart rates. *Isr. J. Zool.* 40, 351-362. 10.1080/00212210.1994.10688759

[JEB247120C6] Burggren, W., Crossley, D., Rogowitz, G. and Thompson, D. (2003). Clutch effects explain heart rate variation in embryonic frogs (Cave Coqui, *Eleutherodactylus cooki*). *Physiol. Biochem. Zool.* 76, 672-678. 10.1086/376918 14671715

[JEB247120C7] Burton, T., Killen, S. S., Armstrong, J. D. and Metcalfe, N. B. (2011). What causes intraspecific variation in resting metabolic rate and what are its ecological consequences? *Proc. R. Soc. B.* 278, 3465-3473. 10.1098/rspb.2011.1778 PMC3189380 21957133

[JEB247120C8] Butler, P. J., Green, J. A., Boyd, I. L. and Speakman, J. R. (2004). Measuring metabolic rate in the field: the pros and cons of the doubly labelled water and heart rate methods. *Funct. Ecol.* 18, 168-183. 10.1111/j.0269-8463.2004.00821.x

[JEB247120C9] Campbell-Staton, S. C., Bare, A., Losos, J. B., Edwards, S. V. and Cheviron, Z. A. (2018). Physiological and regulatory underpinnings of geographic variation in reptilian cold tolerance across a latitudinal cline. *Mol. Ecol.* 27, 2243-2255. 10.1111/mec.14580 29633453

[JEB247120C10] Clark, T. D., Butler, P. J. and Frappell, P. B. (2006). Factors influencing the prediction of metabolic rate in a reptile. *Funct. Ecol.* 20, 105-113. 10.1111/j.1365-2435.2006.01066.x

[JEB247120C11] Cones, A. G. and Westneat, D. F. (2023). Variation in embryonic metabolic reaction norms and the role of the environment. *Physiol. Biochem. Zool.* 96, 260-271. 10.1086/725236 37418603

[JEB247120C12] Cones, A. G., Liebl, A. L., Houslay, T. M. and Russell, A. F. (2021). Temperature-mediated plasticity in incubation schedules is unlikely to evolve to buffer embryos from climatic challenges in a seasonal songbird. *J. Evol. Biol.* 34, 465-476. 10.1111/jeb.13743 33325597

[JEB247120C13] Conway, C. J. and Martin, T. E. (2000). Evolution of passerine incubation behavior: influence of food, temperature, and nest predation. *Evolution* 54, 670-685. 10.1111/j.0014-3820.2000.tb00068.x 10937242

[JEB247120C14] Díaz, J. A., Iraeta, P., Verdú-Ricoy, J., Siliceo, I. and Salvador, A. (2012). Intraspecific variation of reproductive traits in a mediterranean lizard: clutch, population, and lineage effects. *Evol. Biol.* 39, 106-115. 10.1007/s11692-011-9144-5

[JEB247120C15] Du, W. G. and Shine, R. (2015). The behavioural and physiological strategies of bird and reptile embryos in response to unpredictable variation in nest temperature. *Biol. Rev.* 90, 19-30. 10.1111/brv.12089 24593133

[JEB247120C16] Du, W.-G., Radder, R. S., Sun, B. and Shine, R. (2009). Determinants of incubation period: do reptilian embryos hatch after a fixed total number of heart beats? *J. Exp. Biol.* 212, 1302-1306. 10.1242/jeb.027425 19376951

[JEB247120C17] Du, W.-G., Warner, D. A., Langkilde, T., Robbins, T. and Shine, R. (2010). The physiological basis of geographic variation in rates of embryonic development within a widespread lizard species. *Am. Nat.* 176, 522-528. 10.1086/656270 20718676

[JEB247120C18] DuRant, S. E., Hopkins, W. A., Hepp, G. R. and Walters, J. R. (2013). Ecological, evolutionary, and conservation implications of incubation temperature-dependent phenotypes in birds. *Biol. Rev.* 88, 499-509. 10.1111/brv.12015 23368773

[JEB247120C19] Fangue, N. A., Richards, J. G. and Schulte, P. M. (2009). Do mitochondrial properties explain intraspecific variation in thermal tolerance? *J. Exp. Biol.* 212, 514-522. 10.1242/jeb.024034 19181899

[JEB247120C20] Glazier, D. S. (2015a). Body-mass scaling of metabolic rate: what are the relative roles of cellular versus systemic effects? *Biol.* 4, 187-199. 10.3390/biology4010187 PMC4381225 25808601

[JEB247120C21] Glazier, D. S. (2015b). Is metabolic rate a universal ‘pacemaker’ for biological processes? *Biol. Rev.* 90, 377-407. 10.1111/brv.12115 24863680

[JEB247120C22] Gordon, S. P., Hendry, A. P. and Reznick, D. N. (2017). Predator-induced contemporary evolution, phenotypic plasticity, and the evolution of reaction norms in guppies. *Copeia* 105, 514-522. 10.1643/CE-16-522

[JEB247120C23] Green, J. A. (2011). The heart rate method for estimating metabolic rate: review and recommendations. *Comp. Biochem. Physiol.* 158, 287-304. 10.1016/j.cbpa.2010.09.011 20869457

[JEB247120C24] Groothuis, T. G. G., Müller, W., Von Engelhardt, N., Carere, C. and Eising, C. (2005). Maternal hormones as a tool to adjust offspring phenotype in avian species. *Neurosci. Biobehav. Rev.* 29, 329-352. 10.1016/j.neubiorev.2004.12.002 15811503

[JEB247120C25] Hadfield, J. D. (2010). MCMC methods for multi-response generalized linear mixed models: the MCMCglmm R package. *J. Stat. Softw.* 32, 1-22.

[JEB247120C26] Hall, J. M. and Warner, D. A. (2019). Thermal tolerance in the urban heat island: thermal sensitivity varies ontogenetically and differs between embryos of two sympatric ectotherms. *J. Exp. Biol.* 222, jeb210708. 10.1242/jeb.210708 31527177

[JEB247120C27] Jonsson, B., Jonsson, N. and Hansen, M. M. (2022). Knock-on effects of environmental influences during embryonic development of ectothermic vertebrates. *Quart. Rev. Biol.* 97, 95-139. 10.1086/720081

[JEB247120C28] Kar, F., Nakagawa, S., Friesen, C. R. and Noble, D. W. A. (2021). Individual variation in thermal plasticity and its impact on mass-scaling. *Oikos* 130, 1131-1142. 10.1111/oik.08122

[JEB247120C29] Lemoine, N. P. (2019). Moving beyond noninformative priors: why and how to choose weakly informative priors in Bayesian analyses. *Oikos* 128, 912-928. 10.1111/oik.05985

[JEB247120C30] Lighton, J. R. B., Brownell, P. H., Joos, B. and Turner, R. J. (2001). Low metabolic rate in scorpions: implications for population biomass and cannibalism. *J. Exp. Biol.* 204, 607-613. 10.1242/jeb.204.3.607 11171311

[JEB247120C31] Martin, T. E., Ton, R. and Niklison, A. (2013). Intrinsic vs. extrinsic influences on life history expression: metabolism and parentally induced temperature influences on embryo development rate. *Ecol. Lett.* 16, 738-745. 10.1111/ele.12103 23473270

[JEB247120C32] Martin, T. E., Oteyza, J. C., Boyce, A. J., Lloyd, P. and Ton, R. (2015). Adult mortality probability and nest predation rates explain parental effort in warming eggs with consequences for embryonic development time. *Am. Nat.* 186, 223-236. 10.1086/681986 26655151

[JEB247120C33] Nespolo, R. F., Lardies, M. A. and Bozinovic, F. (2003). Intrapopulational variation in the standard metabolic rate of insects: repeatability, thermal dependence and sensitivity (Q_10_) of oxygen consumption in a cricket. *J. Exp. Biol.* 206, 4309-4315. 10.1242/jeb.00687 14581600

[JEB247120C34] Nussey, D. H., Wilson, A. J. and Brommer, J. E. (2007). The evolutionary ecology of individual phenotypic plasticity in wild populations. *J. Evol. Biol.* 20, 831-844. 10.1111/j.1420-9101.2007.01300.x 17465894

[JEB247120C35] Pick, J. L., Kasper, C., Allegue, H., Dingemanse, N. J., Dochtermann, N. A., Laskowski, K. L., Lima, M. R., Schielzeth, H., Westneat, D. F., Wright, J. et al. (2023). Describing posterior distributions of variance components: problems and the use of null distributions to aid interpretation. *Meth. Ecol. Evol.* 14, 2557-2574. 10.1111/2041-210X.14200

[JEB247120C36] Pollard, A. S., Pitsillides, A. A. and Portugal, S. J. (2016). Validating a noninvasive technique for monitoring embryo movement *in ovo*. *Physiol. Biochem. Zool.* 89, 331-339. 10.1086/687228 27327183

[JEB247120C37] Olson, C. R., Vleck, C. M. and Vleck, D. (2006). Periodic cooling of bird eggs reduces embryonic growth efficiency. *Physiol. Biochem. Zool.* 79, 927-936. 10.1086/506003 16927239

[JEB247120C38] Ricklefs, R. and Wikelski, M. (2002). Biodiversity reflects in part the diversification of life histories. *Tree* 17, 462-468. 10.1016/S0169-5347(02)02578-8

[JEB247120C39] Ricklefs, R. E., Austin, S. H. and Robinson, W. D. (2017). The adaptive significance of variation in avian incubation periods. *Auk* 134, 542-550. 10.1642/AUK-16-171.1

[JEB247120C40] Sartori, M. R., Abe, A. S., Crossley, D. A. and Taylor, E. W. (2017). Rates of oxygen uptake increase independently of changes in heart rate in late stages of development and at hatching in the green iguana, *Iguana iguana*. *Comp. Biochem. Physiol.* 205, 28-34. 10.1016/j.cbpa.2016.12.020 28011410

[JEB247120C41] Schaefer, J. and Walters, A. (2010). Metabolic cold adaptation and developmental plasticity in metabolic rates among species in the *Fundulus notatus* species complex. *Funct. Ecol.* 24, 1087-1094. 10.1111/j.1365-2435.2010.01726.x

[JEB247120C42] Schulte, P. M. (2015). The effects of temperature on aerobic metabolism: towards a mechanistic understanding of the responses of ectotherms to a changing environment. *J. Exp. Biol.* 218, 1856-1866. 10.1242/jeb.118851 26085663

[JEB247120C43] Seebacher, F. and Grigaltchik, V. S. (2015). Developmental thermal plasticity of prey modifies the impact of predation. *J. Exp. Biol.* 218, 1402-1409. 10.1242/jeb.116558 25767143

[JEB247120C44] Sheldon, E. L. and Griffith, S. C. (2018). Embryonic heart rate predicts prenatal development rate, but is not related to post-natal growth rate or activity level in the zebra finch (*Taeniopygia guttata*). *Ethology* 124, 829-837. 10.1111/eth.12817

[JEB247120C45] Sheldon, E. L., McCowan, L. S. C., McDiarmid, C. S. and Griffith, S. C. (2018). Measuring the embryonic heart rate of wild birds: an opportunity to take the pulse on early development. *Auk* 135, 71-82. 10.1642/auk-17-111.1

[JEB247120C46] Snell-Rood, E. C. (2013). An overview of the evolutionary causes and consequences of behavioural plasticity. *Anim. Behav.* 85, 1004-1011. 10.1016/j.anbehav.2012.12.031

[JEB247120C47] Sun, B. J., Li, T., Gao, J., Ma, L. and Du, W. G. (2015). High incubation temperatures enhance mitochondrial energy metabolism in reptile embryos. *Sci. Rep.* 5, 8861. 10.1038/srep08861 25749301 PMC4352865

[JEB247120C48] Terblanche, J. S., Janion, C. and Chown, S. L. (2007). Variation in scorpion metabolic rate and rate-temperature relationships: implications for the fundamental equation of the metabolic theory of ecology. *J. Evol. Biol.* 20, 1602-1612. 10.1111/j.1420-9101.2007.01322.x 17584252

[JEB247120C49] Terblanche, J. S., Clusella-Trullas, S., Deere, J. A., Van Vuuren, B. J. and Chown, S. L. (2009). Directional evolution of the slope of the metabolic rate-temperature relationship is correlated with climate. *Physiol. Biochem. Zool.* 82, 495-503. 10.1086/605361 19624273

[JEB247120C50] Tobler, M., Nilsson, J. Å. and Nilsson, J. F. (2007). Costly steroids: egg testosterone modulates nestling metabolic rate in the zebra finch. *Biol. Lett.* 3, 408-410. 10.1098/rsbl.2007.0127 17456447 PMC2390662

[JEB247120C51] Vedder, O., Kürten, N. and Bouwhuis, S. (2017). Intraspecific variation in and environment-dependent resource allocation to embryonic development time in common terns. *Phys. Biochem. Zool.* 90, 453-460. 10.1086/691690 28402235

[JEB247120C52] Webb, D. R. (1987). Thermal tolerances in the avian embryo. *Condor* 89, 874-898. 10.2307/1368537

[JEB247120C53] Westneat, D. F., Potts, L. J., Sasser, K. L. and Shaffer, J. D. (2019). Causes and consequences of phenotypic plasticity in complex environments. *Trends Ecol. Evol.* 34, 555-568. 10.1016/j.tree.2019.02.010 30871734

[JEB247120C54] White, C. R. and Seymour, R. S. (2004). Does basal metabolic rate contain a useful signal? Mammalian BMR allometry and correlations with a selection of physiological, ecological, and life-history variables. *Physiol. Biochem. Zool.* 77, 929-941. 10.1086/425186 15674767

[JEB247120C55] White, C. R., Alton, L. A., Bywater, C. L., Lombardi, E. J. and Marshall, D. J. (2022). Metabolic scaling is the product of life-history optimization. *Science* 377, 834-839. 10.1126/science.abm7649 35981018

[JEB247120C56] Wickham, H. (2016). *ggplot2: elegant graphics for data analysis*. Springer-Verlag New York. https://ggplot2.tidyverse.org.

[JEB247120C57] Woltereck, R. (1909). Weitere experimentelle Untersuchungen über Artveränderung, speziel über das Wesen quantitativer Artunterschiede bei Daphnien. *Verh. Dtsch. Zool. Ges.* 19, 110-173.

[JEB247120C58] Zuo, W., Moses, M. E., West, G. B., Hou, C. and Brown, J. H. (2012). A general model for effects of temperature on ectotherm ontogenetic growth and development. *Proc. R. Soc. B. Biol. Sci.* 279, 1840-1846. 10.1098/rspb.2011.2000 PMC3297449 22130604

