# Supplementary material for: The incubation environment does not explain significant variation in heart rate plasticity among avian embryos
Source: J Exp Biol. 2024 Mar 8;227(5):jeb247120. doi: 10.1242/jeb.247120 (PMC10949066; doi:10.1242/jeb.247120)
Supplement: Supplementary information [file jexbio-227-247120-s1.pdf]

Supplementary Materials and Methods

General set up

First we load the required packages and load our data.

```
library(ggplot2)
library(MCMCglmm)

## Loading required package: Matrix

## Loading required package: coda

## Loading required package: ape

library(tidyr)

##
## Attaching package: 'tidyr'

## The following objects are masked from 'package:Matrix':
##
##     expand, pack, unpack

setwd("~/Desktop/MSRevision")
dat <- read.csv("MSData.csv", header=T)
```

We population-mean-center egg temperature, standardize egg temperature, and split the data into the warm and cooling incubation regime.

```
dat$eggtempc<-scale(dat$eggtemp, scale=F)
dat$eggtempz<-scale(dat$eggtemp, scale=T)
datwarm<-dat[which(dat$incregime=='warm'),]
datcooling<-dat[which(dat$incregime=='cooling'),]
```

Sources of variance in embryonic heart rate reaction norms

We specify weakly informative priors for random effects.

```
prior1<-list(R=list(V=diag(1),nu=0.002),
             G=list(G1=list(V=diag(2), nu=0.002)))
```

We run the random slope model for 100,000 iterations, with a thinning interval of 10, and a burnin of 1,000 iterations. We print out the posterior 95% credible intervals.

Journal of Experimental Biology • Supplementary information

```
heartratemodel<-MCMCglmm(heartrate~eggtempc+incday+incregime+incregime:eggtem
pc,random=~us(1+eggtempc):eggID,nitt=100000,thin=10,burnin=1000,data=dat,prio
r=prior1,verbose=FALSE)
summary(heartratemodel)
```

```
##
##  Iterations = 1001:99991
##  Thinning interval  = 10
##  Sample size  = 9900
##
##  DIC: 4186.126
##
##  G-structure:  ~us(1 + eggtempc):eggID
##
##                                  post.mean  l-95% CI u-95% CI eff.samp
## (Intercept):(Intercept).eggID    93.525   53.267  141.531     9413
## eggtempc:(Intercept).eggID        6.141   -1.584   15.021     9125
## (Intercept):eggtempc.eggID        6.141   -1.584   15.021     9125
## eggtempc:eggtempc.eggID           3.391    1.042    6.048     9757
##
##  R-structure:  ~units
##
##       post.mean l-95% CI u-95% CI eff.samp
## units     44.67    39.41     50.1     9893
##
##  Location effects: heartrate ~ eggtempc + incday + incregime + incregime:e
ggtempc
##
##                          post.mean l-95% CI u-95% CI eff.samp  pMCMC
## (Intercept)               247.0830 239.6938 254.5282    660.9 <1e-04 ***
## eggtempc                   17.5791  16.5114  18.5786   9334.5 <1e-04 ***
## incday                     -2.5268  -2.9063  -2.1412    449.5 <1e-04 ***
## incregimewarm               1.2947  -4.8545   7.5149   9900.0 0.6687
## eggtempc:incregimewarm      1.3265  -0.1259   2.7730   9900.0 0.0723 .
## ---
## Signif. codes:  0 '***' 0.001 '**' 0.01 '*' 0.05 '.' 0.1 ' ' 1
```

We also output the median estimates for the variance terms.

```
apply(heartratemodel$VCV,2,median)
```

```
## (Intercept):(Intercept).eggID    eggtempc:(Intercept).eggID
##                     90.096285                      5.713979
##    (Intercept):eggtempc.eggID       eggtempc:eggtempc.eggID
##                      5.713979                      3.165671
##                         units
##                     44.565056
```

Next, we print the posterior distribution plots.

```
plot(heartratemodel)
```

Journal of Experimental Biology • Supplementary information

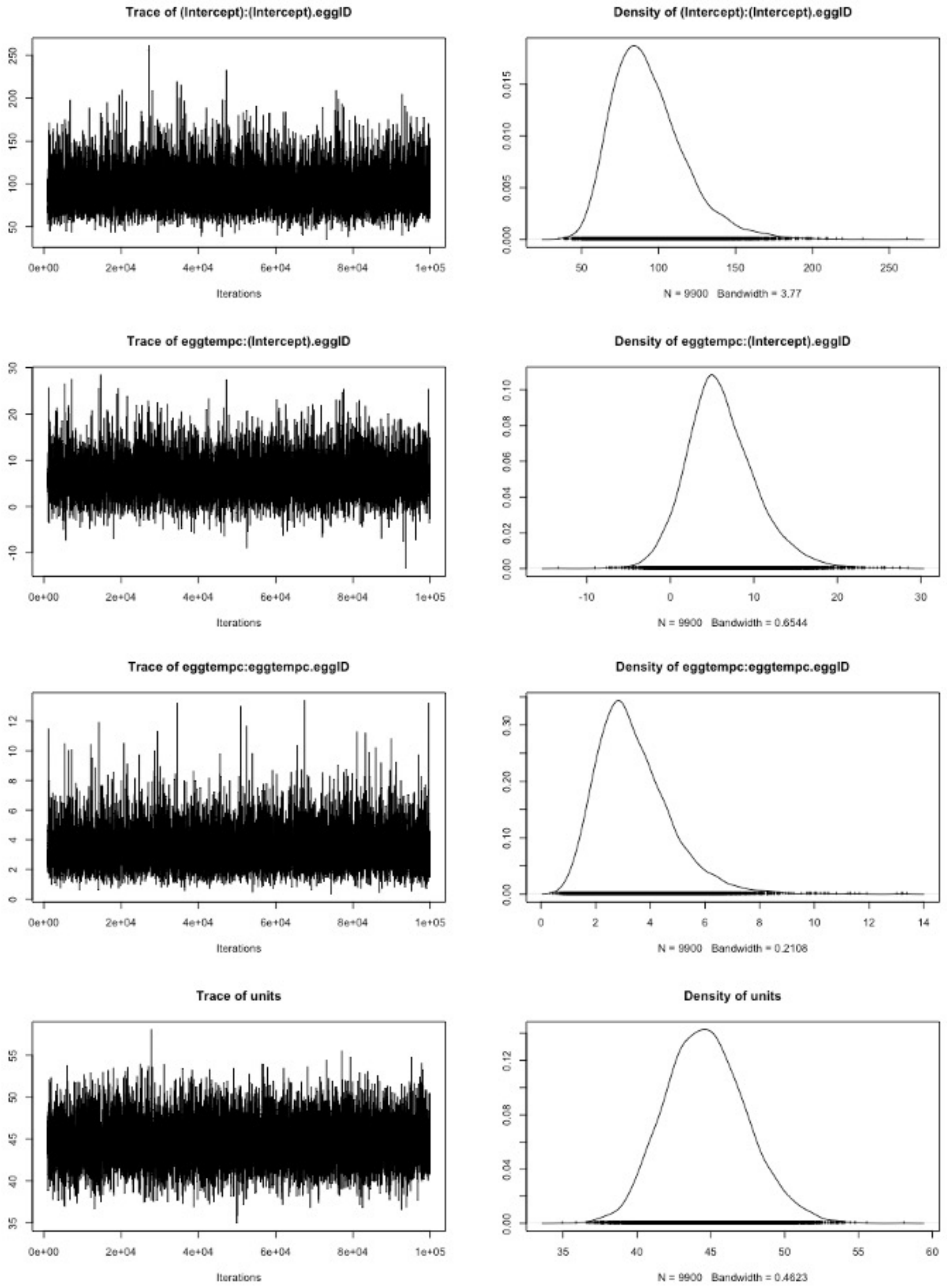

Fig. S1. Posterior distribution of the variance terms in the model.

Journal of Experimental Biology • Supplementary information

Sources of variance in growth

We specify our new priors.

```
prior1b<-list(R=list(V=diag(1),nu=0.002),
             G=list(G1=list(V=diag(1), nu=0.002)))
```

Incubation duration

We test the effect of incubation regime on incubation duration.

```
incdurregime<-MCMCglmm(incdur~incregime,random=~eggID,nitt=100000,thin=10,bur
nin=1000,prior=prior1b,data=dat,verbose=FALSE)
summary(incdurregime)

##
##  Iterations = 1001:99991
##  Thinning interval  = 10
##  Sample size  = 9900
##
##  DIC: -15281.47
##
##  G-structure:  ~eggID
##
##       post.mean l-95% CI u-95% CI eff.samp
## eggID    0.4618   0.2704    0.684     9611
##
##  R-structure:  ~units
##
##       post.mean  l-95% CI  u-95% CI eff.samp
## units 1.544e-06 1.429e-06 1.661e-06    10259
##
##  Location effects: incdur ~ incregime
##
##                post.mean l-95% CI u-95% CI eff.samp   pMCMC
## (Intercept)      28.7208  28.4097  29.0439     9900 < 1e-04 ***
## incregimewarm    -0.6203  -1.0686  -0.1876     9900 0.00929 **
## ---
## Signif. codes:  0 '***' 0.001 '**' 0.01 '*' 0.05 '.' 0.1 ' ' 1
```

Journal of Experimental Biology • Supplementary information

Incubation day heart rate

We perform the same process to test the interaction between embryonic age and incubation regime.

```
heartrateagemodel<-MCMCglmm(heartrate~eggtempc+incday+incregime+incregime:inc
day,random=~us(1+eggtempc):eggID,nitt=100000,thin=10,burnin=1000,data=dat,pri
or=prior1,verbose=FALSE)
summary(heartrateagemodel)

##
##  Iterations = 1001:99991
##  Thinning interval  = 10
##  Sample size  = 9900
##
##  DIC: 4188.41
##
##  G-structure:  ~us(1 + eggtempc):eggID
##
##                                 post.mean l-95% CI u-95% CI eff.samp
## (Intercept):(Intercept).eggID    93.377   52.576  142.278     9900
## eggtempc:(Intercept).eggID        6.730   -1.186   16.353     8783
## (Intercept):eggtempc.eggID        6.730   -1.186   16.353     8783
## eggtempc:eggtempc.eggID           3.787    1.322    6.548     9280
##
##  R-structure:  ~units
##
##       post.mean l-95% CI u-95% CI eff.samp
## units     44.76    39.43    50.21     9190
##
##  Location effects: heartrate ~ eggtempc + incday + incregime + incregime:i
ncday
##
##                        post.mean l-95% CI u-95% CI eff.samp  pMCMC
## (Intercept)             245.6303 235.6830 255.5606    500.4 <1e-04 ***
## eggtempc                 18.2421  17.4748  18.9825   9333.1 <1e-04 ***
## incday                   -2.3815  -2.9803  -1.8302    412.3 <1e-04 ***
## incregimewarm             3.7046  -9.2040  16.5410    585.6  0.590
## incday:incregimewarm     -0.2613  -1.0075   0.4675    462.1  0.503
## ---
## Signif. codes:  0 '***' 0.001 '**' 0.01 '*' 0.05 '.' 0.1 ' ' 1
```

Egg mass

We test the effect of incubation regime on the change in egg mass over the incubation period.

```
eggmassregime<-MCMCglmm(eggmass~incday*incregime,random=~eggID,nitt=100000,th
in=10,burnin=1000,prior=prior1b,data=dat,verbose=FALSE)
summary(eggmassregime)
```

```
##
##  Iterations = 1001:99991
##  Thinning interval  = 10
##  Sample size  = 9900
##
##  DIC: 826.8976
##
##  G-structure:  ~eggID
##
##       post.mean l-95% CI u-95% CI eff.samp
## eggID     20.02    11.87    29.69     9900
##
##  R-structure:  ~units
##
##       post.mean l-95% CI u-95% CI eff.samp
## units    0.4858   0.4086   0.5588     7727
##
##  Location effects: eggmass ~ incday * incregime
##
##                     post.mean l-95% CI u-95% CI eff.samp  pMCMC
## (Intercept)          90.55690 88.59631 92.57947     9900 <1e-04 ***
## incday               -0.25259 -0.26488 -0.24034     6820 <1e-04 ***
## incregimewarm        -0.89730 -3.71058  1.93801     9900  0.517
## incday:incregimewarm -0.04486 -0.06209 -0.02705     6720 <1e-04 ***
## ---
## Signif. codes:  0 '***' 0.001 '**' 0.01 '*' 0.05 '.' 0.1 ' ' 1
```

Duckling tarsus

We test the effect of incubation regime on the growth of duckling tarsus post-hatching.

```
datsubset<-dat[which(dat$ducklingage < 31),]

tarsusregime<-MCMCglmm(tarsus~ducklingage*incregime,random=~eggID,nitt=100000
,thin=10,burnin=1000,prior=prior1b,data=datsubset,verbose=FALSE)
summary(tarsusregime)

##
##  Iterations = 1001:99991
##  Thinning interval  = 10
##  Sample size  = 9900
##
##  DIC: 1941.895
##
##  G-structure:  ~eggID
##
##       post.mean l-95% CI u-95% CI eff.samp
## eggID     3.797     1.82    6.174     5856
##
##  R-structure:  ~units
##
##       post.mean l-95% CI u-95% CI eff.samp
## units     6.144    5.298    7.046     8693
##
##  Location effects: tarsus ~ ducklingage * incregime
##
##                             post.mean l-95% CI u-95% CI eff.samp  pMCMC
## (Intercept)                  23.11855 21.92935 24.38152     2821 <1e-04 ***
## ducklingage                   1.55472  1.51240  1.59739     5790 <1e-04 ***
## incregimewarm                -0.88320 -2.47336  0.69465     3685  0.272
## ducklingage:incregimewarm    -0.01800 -0.07162  0.04130     6762  0.529
## ---
## Signif. codes:  0 '***' 0.001 '**' 0.01 '*' 0.05 '.' 0.1 ' ' 1
```

Journal of Experimental Biology • Supplementary information

Links between heart rate reaction norms and growth

Incubation duration

First, we create our standardized variables, split the data into the warm and cooling incubation regimes.

```
dat$heartratez<-scale(dat$heartrate, scale=T)
dat$eggtempz<-scale(dat$eggtemp, scale=T)
dat$eggmassz<-scale(dat$eggmass, scale=T)
dat$hatchagez<-scale(dat$incdur, scale=T)
dat$tarsusz<-scale(dat$tarsus, scale=T)

datwarm<-dat[which(dat$incregime=='warm'),]
datcooling<-dat[which(dat$incregime=='cooling'),]

datsubset$heartratez<-scale(datsubset$heartrate, scale=T)
datsubset$eggtempz<-scale(datsubset$eggtemp, scale=T)
datsubset$tarsusz<-scale(datsubset$tarsus, scale=T)

datsubsetwarm<-datsubset[which(datsubset$incregime=='warm'),]
datsubsetcooling<-datsubset[which(datsubset$incregime=='cooling'),]
```

First, we estimate the relationship between heart rate reaction norms and incubation duration at the among-individual level.

```
prior3<-list(R=list(V=diag(2),nu=0.002),
             G=list(G1=list(V=diag(3), nu=0.002)))

heartratehatchage<-MCMCglmm(cbind(heartratez,hatchagez)~ trait + at.level(tra
it,1):eggtempz + at.level(trait,1):incday,
                     random=~us(trait+at.level(trait,1):eggtempz):eggID,
                     rcov=~idh(trait):units,
                     family=c("gaussian","gaussian"),
                     nitt=100000,thin=10,burnin=1000,prior=prior3,data=dat,ve
rbose=FALSE)
```

We calculate the estimates as correlations:

Heart rate intercept by incubation duration intercept

```
corR2 <- heartratehatchage$VCV[,"traitheartratez:traithatchagez.eggID"]/
  sqrt(heartratehatchage$VCV[,"traitheartratez:traitheartratez.eggID"]*
       heartratehatchage$VCV[,"traithatchagez:traithatchagez.eggID"])
summary(corR2)

##
## Iterations = 1001:99991
## Thinning interval = 10
## Number of chains = 1
## Sample size per chain = 9900
```

Journal of Experimental Biology • Supplementary information

```
##
## 1. Empirical mean and standard deviation for each variable,
##    plus standard error of the mean:
##
##           Mean             SD       Naive SE Time-series SE
##       0.279330       0.155240       0.001560       0.001629
##
## 2. Quantiles for each variable:
##
##     2.5%      25%      50%      75%    97.5%
## -0.04255  0.17521  0.28706  0.38988  0.56006
```

Heart rate slope by incubation duration intercept

```
corbiv <- heartratehatchage$VCV[,"at.level(trait, 1):eggtempz:traithatchagez.
eggID"]/
  sqrt(heartratehatchage$VCV[,"at.level(trait, 1):eggtempz:at.level(trait, 1)
:eggtempz.eggID"]*
         heartratehatchage$VCV[,"traithatchagez:traithatchagez.eggID"])
summary(corbiv)
```

```
##
## Iterations = 1001:99991
## Thinning interval = 10
## Number of chains = 1
## Sample size per chain = 9900
##
## 1. Empirical mean and standard deviation for each variable,
##    plus standard error of the mean:
##
##           Mean             SD       Naive SE Time-series SE
##      -0.122513       0.196112       0.001971       0.001971
##
## 2. Quantiles for each variable:
##
##      2.5%       25%       50%       75%     97.5%
## -0.491988 -0.260607 -0.125286  0.008604  0.280171
```

Egg mass

Second, we estimate the relationship between heart rate reaction norms and egg mass reaction norms at the among-individual level.

```
prior2<-list(R=list(V=diag(2),nu=0.002),
             G=list(G1=list(V=diag(4), nu=0.002)))

heartratemass<-MCMCglmm(cbind(heartratez,eggmassz)~ trait + at.level(trait,1)
:eggtempz+ at.level(trait,1):incday + at.level(trait,2):incday,
                 random=~us(trait+at.level(trait,1):eggtempz+at.level(trait,2
):incday):eggID,
                 rcov=~us(trait):units,
                 family=c("gaussian","gaussian"),
                 nitt=100000,thin=10,burnin=1000,prior=prior2,data=dat,verbos
e=FALSE)
```

We calculate the estimates as correlations:

Heart rate intercept by egg mass intercept

```
corR2 <- heartratemass$VCV[,"traitheartratez:traiteggmassz.eggID"]/
  sqrt(heartratemass$VCV[,"traitheartratez:traitheartratez.eggID"]*
       heartratemass$VCV[,"traiteggmassz:traiteggmassz.eggID"])
summary(corR2)

##
## Iterations = 1001:99991
## Thinning interval = 10
## Number of chains = 1
## Sample size per chain = 9900
##
## 1. Empirical mean and standard deviation for each variable,
##    plus standard error of the mean:
##
##           Mean             SD       Naive SE Time-series SE
##      -0.121823       0.165020       0.001659       0.001659
##
## 2. Quantiles for each variable:
##
##     2.5%      25%      50%      75%    97.5%
## -0.43290 -0.23677 -0.12483 -0.01021  0.21128
```

Heart rate intercept by egg mass slope

```
corR2 <- heartratemass$VCV[,"traitheartratez:at.level(trait, 2):incday.eggID"
]/
  sqrt(heartratemass$VCV[,"traitheartratez:traitheartratez.eggID"]*
       heartratemass$VCV[,"at.level(trait, 2):incday:at.level(trait, 2):inc
day.eggID"])
summary(corR2)
```

Journal of Experimental Biology • Supplementary information

```
##
## Iterations = 1001:99991
## Thinning interval = 10
## Number of chains = 1
## Sample size per chain = 9900
##
## 1. Empirical mean and standard deviation for each variable,
##    plus standard error of the mean:
##
##          Mean             SD       Naive SE Time-series SE
##       0.416228       0.141225       0.001419       0.001419
##
## 2. Quantiles for each variable:
##
##   2.5%    25%    50%    75%  97.5%
## 0.1150 0.3262 0.4264 0.5175 0.6605
```

Heart rate slope by egg mass intercept

```
corbiv <- heartratemass$VCV[,"at.level(trait, 1):eggtempz:traiteggmassz.eggID
"]/
  sqrt(heartratemass$VCV[,"at.level(trait, 1):eggtempz:at.level(trait, 1):egg
tempz.eggID"]*
         heartratemass$VCV[,"traiteggmassz:traiteggmassz.eggID"])
summary(corbiv)
```

```
##
## Iterations = 1001:99991
## Thinning interval = 10
## Number of chains = 1
## Sample size per chain = 9900
##
## 1. Empirical mean and standard deviation for each variable,
##    plus standard error of the mean:
##
##          Mean             SD       Naive SE Time-series SE
##      -0.206476       0.193147       0.001941       0.002356
##
## 2. Quantiles for each variable:
##
##     2.5%      25%      50%      75%    97.5%
## -0.56091 -0.34501 -0.21155 -0.07762  0.18350
```

Heart rate slope by egg mass slope

```
corbiv <- heartratemass$VCV[,"at.level(trait, 1):eggtempz:at.level(trait, 2):
incday.eggID"]/
  sqrt(heartratemass$VCV[,"at.level(trait, 1):eggtempz:at.level(trait, 1):egg
tempz.eggID"]*
         heartratemass$VCV[,"at.level(trait, 2):incday:at.level(trait, 2):inc
```

Journal of Experimental Biology • Supplementary information

```
day.eggID"])
summary(corbiv)

##
## Iterations = 1001:99991
## Thinning interval = 10
## Number of chains = 1
## Sample size per chain = 9900
##
## 1. Empirical mean and standard deviation for each variable,
##    plus standard error of the mean:
##
##           Mean             SD       Naive SE Time-series SE
##      -0.075679       0.187788       0.001887       0.002237
##
## 2. Quantiles for each variable:
##
##     2.5%      25%      50%      75%    97.5%
## -0.43477 -0.20554 -0.07867  0.05251  0.29791
```

Journal of Experimental Biology • Supplementary information

Duckling tarsus

Third, we estimate the relationship between heart rate reaction norms and tarsus reaction norms at the among-individual level.

```
prior2<-list(R=list(V=diag(2),nu=0.002),
             G=list(G1=list(V=diag(4), nu=0.002)))

heartratetarsus<-MCMCglmm(cbind(heartratez,tarsusz)~ trait + at.level(trait,1
):eggtempz+ at.level(trait,1):incday + at.level(trait,2):ducklingage,
                   random=~us(trait+at.level(trait,1):eggtempz+at.level(trait
,2):ducklingage):eggID,
                   rcov=~us(trait):units,
                   family=c("gaussian","gaussian"),
                   nitt=100000,thin=10,burnin=1000,prior=prior2,data=datsubse
t,verbose=FALSE)
```

We calculate the estimates as correlations:

Heart rate intercept by tarsus intercept

```
corR2 <- heartratetarsus$VCV[,"traitheartratez:traittarsusz.eggID"]/
  sqrt(heartratetarsus$VCV[,"traitheartratez:traitheartratez.eggID"]*
         heartratetarsus$VCV[,"traittarsusz:traittarsusz.eggID"])
summary(corR2)

##
## Iterations = 1001:99991
## Thinning interval = 10
## Number of chains = 1
## Sample size per chain = 9900
##
## 1. Empirical mean and standard deviation for each variable,
##    plus standard error of the mean:
##
##           Mean             SD       Naive SE Time-series SE
##      -0.776232       0.138789       0.001395       0.006345
##
## 2. Quantiles for each variable:
##
##    2.5%     25%     50%     75%   97.5%
## -0.9580 -0.8768 -0.8057 -0.7071 -0.4187
```

Heart rate intercept by tarsus slope

```
corR2 <- heartratetarsus$VCV[,"traitheartratez:at.level(trait, 2):ducklingage
.eggID"]/
  sqrt(heartratetarsus$VCV[,"traitheartratez:traitheartratez.eggID"]*
         heartratetarsus$VCV[,"at.level(trait, 2):ducklingage:at.level(trait,
2):ducklingage.eggID"])
summary(corR2)
```

Journal of Experimental Biology • Supplementary information

```
##
## Iterations = 1001:99991
## Thinning interval = 10
## Number of chains = 1
## Sample size per chain = 9900
##
## 1. Empirical mean and standard deviation for each variable,
##    plus standard error of the mean:
##
##           Mean             SD       Naive SE Time-series SE
##       -0.024337       0.227734       0.002289       0.005146
##
## 2. Quantiles for each variable:
##
##     2.5%      25%      50%      75%    97.5%
## -0.45292 -0.18594 -0.02678  0.13526  0.41977
```

Heart rate slope by tarsus intercept

```
corbiv <- heartratetarsus$VCV[,"at.level(trait, 1):eggtempz:traittarsusz.eggI
D"]/
  sqrt(heartratetarsus$VCV[,"at.level(trait, 1):eggtempz:at.level(trait, 1):e
ggtempz.eggID"]*
         heartratetarsus$VCV[,"traittarsusz:traittarsusz.eggID"])
summary(corbiv)
```

```
##
## Iterations = 1001:99991
## Thinning interval = 10
## Number of chains = 1
## Sample size per chain = 9900
##
## 1. Empirical mean and standard deviation for each variable,
##    plus standard error of the mean:
##
##           Mean             SD       Naive SE Time-series SE
##       -0.119546       0.273896       0.002753       0.013301
##
## 2. Quantiles for each variable:
##
##     2.5%      25%      50%      75%    97.5%
## -0.61779 -0.31678 -0.12777  0.06987  0.42889
```

Heart rate slope by tarsus slope

```
corbiv <- heartratetarsus$VCV[,"at.level(trait, 1):eggtempz:at.level(trait, 2
):ducklingage.eggID"]/
  sqrt(heartratetarsus$VCV[,"at.level(trait, 1):eggtempz:at.level(trait, 1):e
ggtempz.eggID"]*
         heartratetarsus$VCV[,"at.level(trait, 2):ducklingage:at.level(trait,
```

```
2):ducklingage.eggID"])
summary(corbiv)

##
## Iterations = 1001:99991
## Thinning interval = 10
## Number of chains = 1
## Sample size per chain = 9900
##
## 1. Empirical mean and standard deviation for each variable,
##    plus standard error of the mean:
##
##           Mean             SD       Naive SE Time-series SE
##       0.101302       0.218988       0.002201       0.004624
##
## 2. Quantiles for each variable:
##
##     2.5%      25%      50%      75%    97.5%
## -0.33655 -0.04922  0.10704  0.25716  0.50758
```

Effects of incubation regime on (co)variance of heart rate reaction norms and growth – warm regime

We estimate variance terms in the warm incubation regime.

```
heartratemodelwarm<-MCMCglmm(heartrate~eggtempc+incday,random=~us(1+eggtempc)
:eggID,nitt=100000,thin=10,burnin=1000,data=datwarm,prior=prior1,verbose=FALS
E)
summary(heartratemodelwarm)
##  Iterations = 1001:99991
##  Thinning interval  = 10
##  Sample size  = 9900
##
##  DIC: 2147.319
##
##  G-structure:  ~us(1 + eggtempc):eggID
##
##                                post.mean   l-95% CI u-95% CI eff.samp
## (Intercept):(Intercept).eggID   124.787 51.8969233  222.816     9688
## eggtempc:(Intercept).eggID        9.168 -4.7174014   27.368     6026
## (Intercept):eggtempc.eggID        9.168 -4.7174014   27.368     6026
## eggtempc:eggtempc.eggID           4.584  0.0005217    9.631     2531
##
##  R-structure:  ~units
##
##       post.mean l-95% CI u-95% CI eff.samp
## units     46.01    38.37       54     4579
##
##  Location effects: heartrate ~ eggtempc + incday
##
##             post.mean l-95% CI u-95% CI eff.samp  pMCMC
## (Intercept)   250.126  240.896  259.468    633.2 <1e-04 ***
## eggtempc       18.920   17.794   20.067   9900.0 <1e-04 ***
## incday         -2.644   -3.152   -2.131    457.6 <1e-04 ***
## ---
## Signif. codes:  0 '***' 0.001 '**' 0.01 '*' 0.05 '.' 0.1 ' ' 1
```

We output the median estimates.

```
apply(heartratemodelwarm$VCV,2,median)

## (Intercept):(Intercept).eggID    eggtempc:(Intercept).eggID
##                    114.932507                      8.034978
##    (Intercept):eggtempc.eggID       eggtempc:eggtempc.eggID
##                      8.034978                      4.102049
##                         units
##                     45.777081
```

Journal of Experimental Biology • Supplementary information

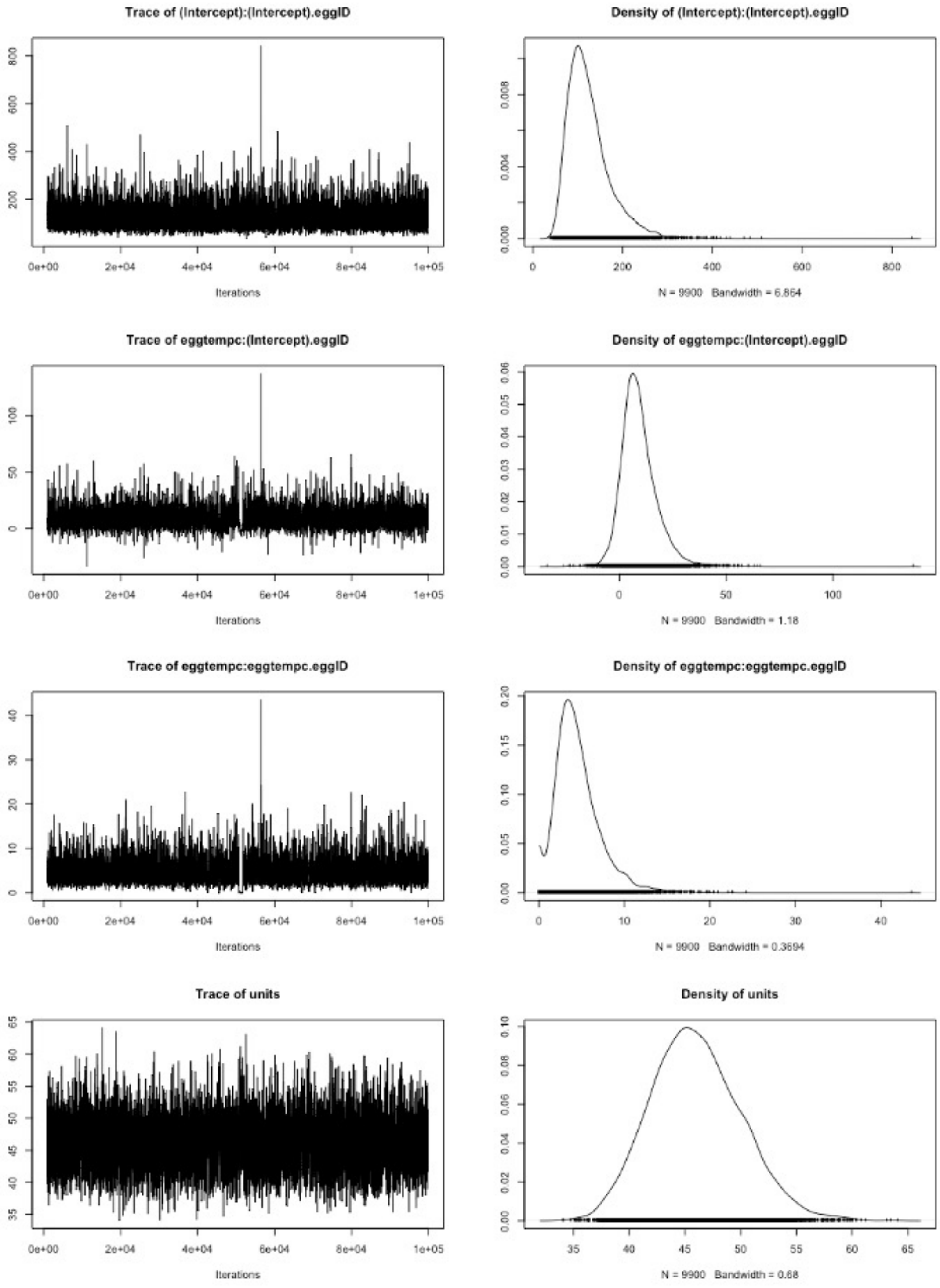

Fig. S2. Posterior distribution of the variance terms in the constant warm regime.

Journal of Experimental Biology • Supplementary information

Effects of incubation regime on (co)variance of heart rate reaction norms and growth – cooling regime

We estimate variance terms in the cooling incubation regime.

```
heartratemodelcooling<-MCMCglmm(heartrate~eggtempc+incday,random=~us(1+eggtem
pc):eggID,nitt=100000,thin=10,burnin=1000,data=datcooling,prior=prior1,verbos
e=FALSE)
summary(heartratemodelcooling)
##  Iterations = 1001:99991
##  Thinning interval  = 10
##  Sample size  = 9900
##
##  DIC: 2054.777
##
##  G-structure:  ~us(1 + eggtempc):eggID
##
##                               post.mean   l-95% CI  u-95% CI eff.samp
## (Intercept):(Intercept).eggID    76.875 31.3495140  137.548   9900.0
## eggtempc:(Intercept).eggID        2.785 -6.0762293   12.841    566.0
## (Intercept):eggtempc.eggID        2.785 -6.0762293   12.841    566.0
## eggtempc:eggtempc.eggID           1.157  0.0002404    4.048    671.1
##
##  R-structure:  ~units
##
##       post.mean l-95% CI u-95% CI eff.samp
## units     46.11    38.32    54.56     1691
##
##  Location effects: heartrate ~ eggtempc + incday
##
##             post.mean l-95% CI u-95% CI eff.samp  pMCMC
## (Intercept)   244.312  234.673  253.783    583.5 <1e-04 ***
## eggtempc       17.390   16.593   18.248   4264.7 <1e-04 ***
## incday         -2.357   -2.903   -1.811    471.7 <1e-04 ***
## ---
## Signif. codes:  0 '***' 0.001 '**' 0.01 '*' 0.05 '.' 0.1 ' ' 1
```

We output the median estimates.

```
apply(heartratemodelcooling$VCV,2,median)

## (Intercept):(Intercept).eggID    eggtempc:(Intercept).eggID
##                    70.4912480                     2.2582400
##    (Intercept):eggtempc.eggID       eggtempc:eggtempc.eggID
##                     2.2582400                     0.5643601
##                         units
##                    45.9429873
```

Journal of Experimental Biology • Supplementary information

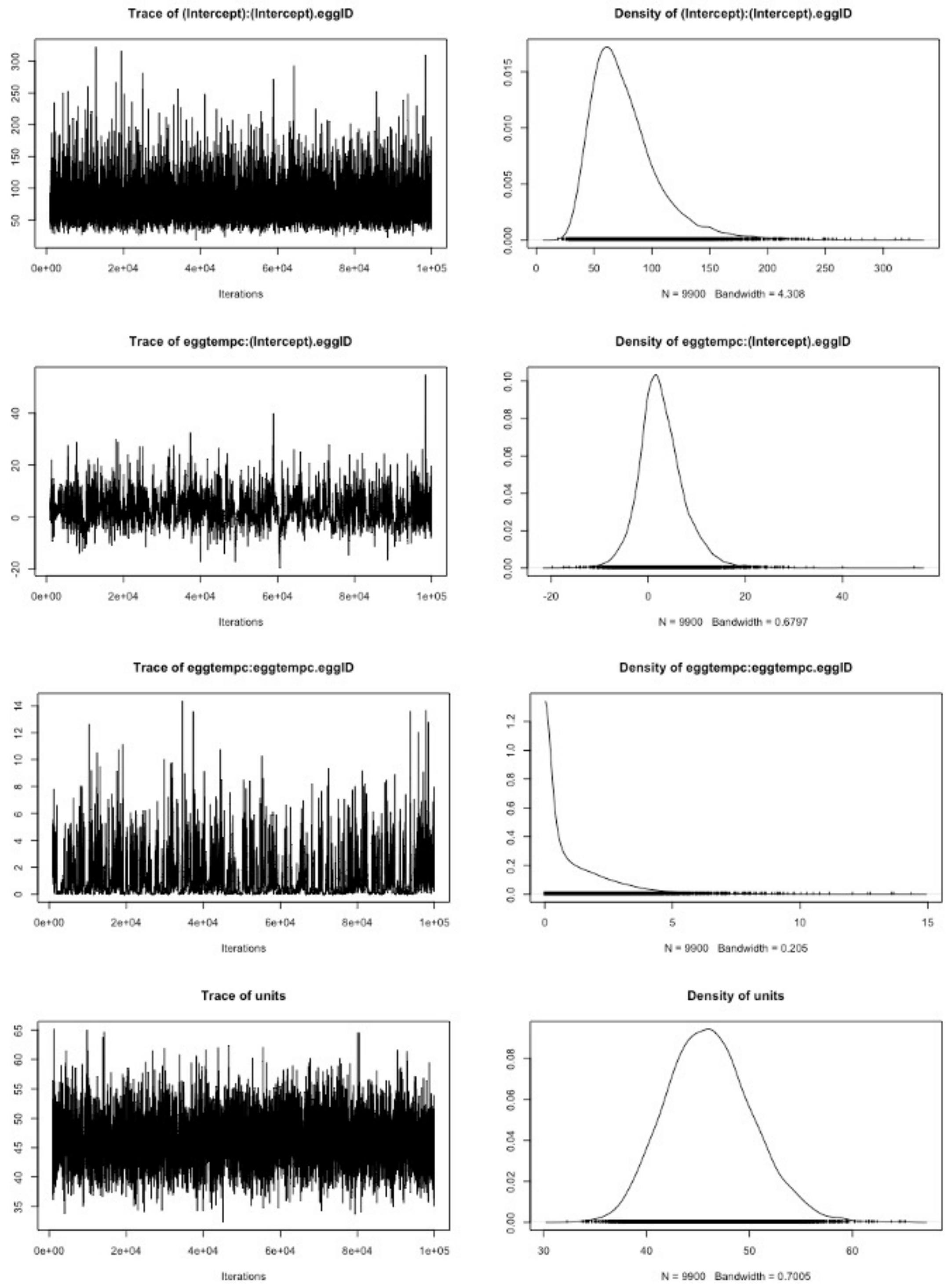

Fig. S3. Posterior distribution of the variance terms in the variable cooling regime.

Journal of Experimental Biology • Supplementary information

Effects of incubation regime on covariance - warm incubation regime

Incubation duration

First, we estimate the relationship between heart rate reaction norms and incubation duration at the among-individual level.

```
prior3<-list(R=list(V=diag(2),nu=0.002),
             G=list(G1=list(V=diag(3), nu=0.002)))

heartratehatchagew<-MCMCglmm(cbind(heartratez,hatchagez)~ trait + at.level(tr
ait,1):eggtempz + at.level(trait,1):incday,
                     random=~us(trait+at.level(trait,1):eggtempz):eggID,
                     rcov=~idh(trait):units,
                     family=c("gaussian","gaussian"),
                     nitt=100000,thin=10,burnin=1000,prior=prior3,data=datwar
m,verbose=FALSE)
```

We calculate the estimates as correlations:

Heart rate intercept by incubation duration intercept

```
corR2 <- heartratehatchagew$VCV[,"traitheartratez:traithatchagez.eggID"]/
  sqrt(heartratehatchagew$VCV[,"traitheartratez:traitheartratez.eggID"]*
         heartratehatchagew$VCV[,"traithatchagez:traithatchagez.eggID"])
summary(corR2)

##
## Iterations = 1001:99991
## Thinning interval = 10
## Number of chains = 1
## Sample size per chain = 9900
##
## 1. Empirical mean and standard deviation for each variable,
##    plus standard error of the mean:
##
##           Mean             SD       Naive SE Time-series SE
##       0.457123       0.194010       0.001950       0.002201
##
## 2. Quantiles for each variable:
##
##    2.5%     25%     50%     75%   97.5%
## 0.02438 0.33716 0.47952 0.59983 0.77219
```

Heart rate slope by incubation duration intercept

```
corbiv <- heartratehatchagew$VCV[,"at.level(trait, 1):eggtempz:traithatchagez
.eggID"]/
  sqrt(heartratehatchagew$VCV[,"at.level(trait, 1):eggtempz:at.level(trait, 1
):eggtempz.eggID"]*
```

```
        heartratehatchagew$VCV[,"traithatchagez:traithatchagez.eggID"])
summary(corbiv)

##
## Iterations = 1001:99991
## Thinning interval = 10
## Number of chains = 1
## Sample size per chain = 9900
##
## 1. Empirical mean and standard deviation for each variable,
##    plus standard error of the mean:
##
##           Mean             SD       Naive SE Time-series SE
##       0.142434       0.275306       0.002767       0.002767
##
## 2. Quantiles for each variable:
##
##     2.5%      25%      50%      75%    97.5%
## -0.41237 -0.04682  0.15156  0.34125  0.64474
```

Egg mass

Second, we estimate the relationship between heart rate reaction norms and egg mass reaction norms at the among-individual level.

```
prior2<-list(R=list(V=diag(2),nu=0.002),
             G=list(G1=list(V=diag(4), nu=0.002)))

heartratemassw<-MCMCglmm(cbind(heartratez,eggmassz)~ trait + at.level(trait,1
):eggtempz+ at.level(trait,1):incday + at.level(trait,2):incday,
                 random=~us(trait+at.level(trait,1):eggtempz+at.level(trait,2
):incday):eggID,
                 rcov=~us(trait):units,
                 family=c("gaussian","gaussian"),
                 nitt=100000,thin=10,burnin=1000,prior=prior2,data=datwarm,ve
rbose=FALSE)
```

We calculate the estimates as correlations:

Heart rate intercept by egg mass intercept

```
corR2 <- heartratemassw$VCV[,"traitheartratez:traiteggmassz.eggID"]/
  sqrt(heartratemassw$VCV[,"traitheartratez:traitheartratez.eggID"]*
       heartratemassw$VCV[,"traiteggmassz:traiteggmassz.eggID"])
summary(corR2)

##
## Iterations = 1001:99991
## Thinning interval = 10
## Number of chains = 1
## Sample size per chain = 9900
##
## 1. Empirical mean and standard deviation for each variable,
##    plus standard error of the mean:
##
##           Mean             SD       Naive SE Time-series SE
##      -0.178398       0.238026       0.002392       0.002479
##
## 2. Quantiles for each variable:
##
##     2.5%      25%      50%      75%    97.5%
## -0.61169 -0.34879 -0.18783 -0.01643  0.31247
```

Heart rate intercept by egg mass slope

```
corR2 <- heartratemassw$VCV[,"traitheartratez:at.level(trait, 2):incday.eggID
"]/
  sqrt(heartratemassw$VCV[,"traitheartratez:traitheartratez.eggID"]*
       heartratemassw$VCV[,"at.level(trait, 2):incday:at.level(trait, 2):in
cday.eggID"])
summary(corR2)
```

Journal of Experimental Biology • Supplementary information

```
##
## Iterations = 1001:99991
## Thinning interval = 10
## Number of chains = 1
## Sample size per chain = 9900
##
## 1. Empirical mean and standard deviation for each variable,
##    plus standard error of the mean:
##
##           Mean             SD       Naive SE Time-series SE
##       0.568905       0.173582       0.001745       0.001745
##
## 2. Quantiles for each variable:
##
##   2.5%    25%    50%    75%  97.5%
## 0.1591 0.4661 0.5946 0.6967 0.8318
```

Heart rate slope by egg mass intercept

```
corbiv <- heartratemassw$VCV[,"at.level(trait, 1):eggtempz:traiteggmassz.eggI
D"]/
  sqrt(heartratemassw$VCV[,"at.level(trait, 1):eggtempz:at.level(trait, 1):eg
gtempz.eggID"]*
         heartratemassw$VCV[,"traiteggmassz:traiteggmassz.eggID"])
summary(corbiv)
```

```
##
## Iterations = 1001:99991
## Thinning interval = 10
## Number of chains = 1
## Sample size per chain = 9900
##
## 1. Empirical mean and standard deviation for each variable,
##    plus standard error of the mean:
##
##           Mean             SD       Naive SE Time-series SE
##      -0.159158       0.283305       0.002847       0.003934
##
## 2. Quantiles for each variable:
##
##    2.5%     25%     50%     75%   97.5%
## -0.6645 -0.3702 -0.1703  0.0381  0.4155
```

Heart rate slope by egg mass slope

```
corbiv <- heartratemassw$VCV[,"at.level(trait, 1):eggtempz:at.level(trait, 2)
:incday.eggID"]/
  sqrt(heartratemassw$VCV[,"at.level(trait, 1):eggtempz:at.level(trait, 1):eg
gtempz.eggID"]*
         heartratemassw$VCV[,"at.level(trait, 2):incday:at.level(trait, 2):in
```

Journal of Experimental Biology • Supplementary information

```
cday.eggID"])
summary(corbiv)

##
## Iterations = 1001:99991
## Thinning interval = 10
## Number of chains = 1
## Sample size per chain = 9900
##
## 1. Empirical mean and standard deviation for each variable,
##    plus standard error of the mean:
##
##           Mean             SD       Naive SE Time-series SE
##       0.162092       0.263672       0.002650       0.003294
##
## 2. Quantiles for each variable:
##
##     2.5%      25%      50%      75%    97.5%
## -0.36907 -0.01914  0.17485  0.35412  0.63476
```

Duckling tarsus

Third, we estimate the relationship between heart rate reaction norms and tarsus reaction norms at the among-individual level

```
prior2<-list(R=list(V=diag(2),nu=0.002),
             G=list(G1=list(V=diag(4), nu=0.002)))

heartratetarsusw<-MCMCglmm(cbind(heartratez,tarsusz)~ trait + at.level(trait,
1):eggtempz+ at.level(trait,1):incday + at.level(trait,2):ducklingage,
                   random=~us(trait+at.level(trait,1):eggtempz+at.level(trait
,2):ducklingage):eggID,
                   rcov=~us(trait):units,
                   family=c("gaussian","gaussian"),
                   nitt=100000,thin=10,burnin=1000,prior=prior2,data=datsubse
twarm,verbose=FALSE)
```

We calculate the estimates as correlations:

Heart rate intercept by tarsus intercept

```
corR2 <- heartratetarsusw$VCV[,"traitheartratez:traittarsusz.eggID"]/
  sqrt(heartratetarsusw$VCV[,"traitheartratez:traitheartratez.eggID"]*
         heartratetarsusw$VCV[,"traittarsusz:traittarsusz.eggID"])
summary(corR2)

##
## Iterations = 1001:99991
## Thinning interval = 10
## Number of chains = 1
## Sample size per chain = 9900
##
## 1. Empirical mean and standard deviation for each variable,
##    plus standard error of the mean:
##
##           Mean             SD       Naive SE Time-series SE
##      -0.844906       0.142296       0.001430       0.008808
##
## 2. Quantiles for each variable:
##
##    2.5%     25%     50%     75%   97.5%
## -0.9827 -0.9413 -0.8888 -0.7966 -0.4412
```

Heart rate intercept by tarsus slope

```
corR2 <- heartratetarsusw$VCV[,"traitheartratez:at.level(trait, 2):ducklingag
e.eggID"]/
  sqrt(heartratetarsusw$VCV[,"traitheartratez:traitheartratez.eggID"]*
         heartratetarsusw$VCV[,"at.level(trait, 2):ducklingage:at.level(trait
, 2):ducklingage.eggID"])
summary(corR2)
```

Journal of Experimental Biology • Supplementary information

```
##
## Iterations = 1001:99991
## Thinning interval = 10
## Number of chains = 1
## Sample size per chain = 9900
##
## 1. Empirical mean and standard deviation for each variable,
##    plus standard error of the mean:
##
##           Mean             SD       Naive SE Time-series SE
##       0.014955       0.307317       0.003089       0.005221
##
## 2. Quantiles for each variable:
##
##     2.5%      25%      50%      75%    97.5%
## -0.57093 -0.20973  0.01449  0.24042  0.59396
```

Heart rate slope by tarsus intercept

```
corbiv <- heartratetarsusw$VCV[,"at.level(trait, 1):eggtempz:traittarsusz.egg
ID"]/
  sqrt(heartratetarsusw$VCV[,"at.level(trait, 1):eggtempz:at.level(trait, 1):
eggtempz.eggID"]*
         heartratetarsusw$VCV[,"traittarsusz:traittarsusz.eggID"])
summary(corbiv)
```

```
##
## Iterations = 1001:99991
## Thinning interval = 10
## Number of chains = 1
## Sample size per chain = 9900
##
## 1. Empirical mean and standard deviation for each variable,
##    plus standard error of the mean:
##
##           Mean             SD       Naive SE Time-series SE
##      -0.298862       0.308089       0.003096       0.009188
##
## 2. Quantiles for each variable:
##
##     2.5%      25%      50%      75%    97.5%
## -0.80124 -0.53338 -0.33061 -0.09589  0.36737
```

Heart rate slope by tarsus slope

```
corbiv <- heartratetarsusw$VCV[,"at.level(trait, 1):eggtempz:at.level(trait,
2):ducklingage.eggID"]/
  sqrt(heartratetarsusw$VCV[,"at.level(trait, 1):eggtempz:at.level(trait, 1):
eggtempz.eggID"]*
         heartratetarsusw$VCV[,"at.level(trait, 2):ducklingage:at.level(trait
```

Journal of Experimental Biology • Supplementary information

```
, 2):ducklingage.eggID"])
summary(corbiv)

##
## Iterations = 1001:99991
## Thinning interval = 10
## Number of chains = 1
## Sample size per chain = 9900
##
## 1. Empirical mean and standard deviation for each variable,
##    plus standard error of the mean:
##
##           Mean             SD       Naive SE Time-series SE
##       0.197084       0.268454       0.002698       0.004849
##
## 2. Quantiles for each variable:
##
##     2.5%      25%      50%      75%    97.5%
## -0.35544  0.01544  0.21196  0.39590  0.66945
```

Journal of Experimental Biology • Supplementary information

Effects of incubation regime on covariance - cooling incubation regime

Incubation duration

First, we estimate the relationship between heart rate reaction norms and incubation duration at the among-individual level.

```
prior3<-list(R=list(V=diag(2),nu=0.002),
             G=list(G1=list(V=diag(3), nu=0.002)))

heartratehatchagec<-MCMCglmm(cbind(heartratez,hatchagez)~ trait + at.level(tr
ait,1):eggtempz + at.level(trait,1):incday,
                     random=~us(trait+at.level(trait,1):eggtempz):eggID,
                     rcov=~idh(trait):units,
                     family=c("gaussian","gaussian"),
                     nitt=100000,thin=10,burnin=1000,prior=prior3,data=datcoo
ling,verbose=FALSE)
```

We calculate the estimates as correlations:

Heart rate intercept by incubation duration intercept

```
corR2 <- heartratehatchagec$VCV[,"traitheartratez:traithatchagez.eggID"]/
  sqrt(heartratehatchagec$VCV[,"traitheartratez:traitheartratez.eggID"]*
         heartratehatchagec$VCV[,"traithatchagez:traithatchagez.eggID"])
summary(corR2)

##
## Iterations = 1001:99991
## Thinning interval = 10
## Number of chains = 1
## Sample size per chain = 9900
##
## 1. Empirical mean and standard deviation for each variable,
##    plus standard error of the mean:
##
##           Mean             SD       Naive SE Time-series SE
##      -0.060563       0.239623       0.002408       0.002408
##
## 2. Quantiles for each variable:
##
##     2.5%      25%      50%      75%    97.5%
## -0.52011 -0.23271 -0.06407  0.10679  0.40940
```

Heart rate slope by incubation duration intercept

```
corbiv <- heartratehatchagec$VCV[,"at.level(trait, 1):eggtempz:traithatchagez
.eggID"]/
  sqrt(heartratehatchagec$VCV[,"at.level(trait, 1):eggtempz:at.level(trait, 1
):eggtempz.eggID"]*
```

Journal of Experimental Biology • Supplementary information

```
         heartratehatchagec$VCV[,"traithatchagez:traithatchagez.eggID"])
summary(corbiv)

##
## Iterations = 1001:99991
## Thinning interval = 10
## Number of chains = 1
## Sample size per chain = 9900
##
## 1. Empirical mean and standard deviation for each variable,
##    plus standard error of the mean:
##
##           Mean             SD       Naive SE Time-series SE
##       -0.411299       0.289584       0.002910       0.003194
##
## 2. Quantiles for each variable:
##
##    2.5%     25%     50%     75%   97.5%
## -0.8548 -0.6311 -0.4502 -0.2270  0.2335
```

Egg mass

Second, we estimate the relationship between heart rate reaction norms and egg mass reaction norms at the among-individual level.

```
prior2<-list(R=list(V=diag(2),nu=0.002),
             G=list(G1=list(V=diag(4), nu=0.002)))

heartratemassc<-MCMCglmm(cbind(heartratez,eggmassz)~ trait + at.level(trait,1
):eggtempz+ at.level(trait,1):incday + at.level(trait,2):incday,
                 random=~us(trait+at.level(trait,1):eggtempz+at.level(trait,2
):incday):eggID,
                 rcov=~us(trait):units,
                 family=c("gaussian","gaussian"),
                 nitt=100000,thin=10,burnin=1000,prior=prior2,data=datcooling
,verbose=FALSE)
```

We calculate the estimates as correlations:

Heart rate intercept by egg mass intercept

```
corR2 <- heartratemassc$VCV[,"traitheartratez:traiteggmassz.eggID"]/
  sqrt(heartratemassc$VCV[,"traitheartratez:traitheartratez.eggID"]*
       heartratemassc$VCV[,"traiteggmassz:traiteggmassz.eggID"])
summary(corR2)

##
## Iterations = 1001:99991
## Thinning interval = 10
## Number of chains = 1
## Sample size per chain = 9900
##
## 1. Empirical mean and standard deviation for each variable,
##    plus standard error of the mean:
##
##           Mean             SD       Naive SE Time-series SE
##      -0.042324       0.248867       0.002501       0.002569
##
## 2. Quantiles for each variable:
##
##     2.5%      25%      50%      75%    97.5%
## -0.50988 -0.22218 -0.04583  0.13215  0.43972
```

Heart rate intercept by egg mass slope

```
corR2 <- heartratemassc$VCV[,"traitheartratez:at.level(trait, 2):incday.eggID
"]/
  sqrt(heartratemassc$VCV[,"traitheartratez:traitheartratez.eggID"]*
       heartratemassc$VCV[,"at.level(trait, 2):incday:at.level(trait, 2):in
cday.eggID"])
summary(corR2)
```

```
##
## Iterations = 1001:99991
## Thinning interval = 10
## Number of chains = 1
## Sample size per chain = 9900
##
## 1. Empirical mean and standard deviation for each variable,
##    plus standard error of the mean:
##
##           Mean             SD       Naive SE Time-series SE
##       0.122281       0.243368       0.002446       0.002446
##
## 2. Quantiles for each variable:
##
##     2.5%      25%      50%      75%    97.5%
## -0.35991 -0.04491  0.12956  0.30016  0.57007
```

Heart rate slope by egg mass intercept

```
corbiv <- heartratemassc$VCV[,"at.level(trait, 1):eggtempz:traiteggmassz.eggI
D"]/
  sqrt(heartratemassc$VCV[,"at.level(trait, 1):eggtempz:at.level(trait, 1):eg
gtempz.eggID"]*
         heartratemassc$VCV[,"traiteggmassz:traiteggmassz.eggID"])
summary(corbiv)
```

```
##
## Iterations = 1001:99991
## Thinning interval = 10
## Number of chains = 1
## Sample size per chain = 9900
##
## 1. Empirical mean and standard deviation for each variable,
##    plus standard error of the mean:
##
##           Mean             SD       Naive SE Time-series SE
##      -0.187935       0.307655       0.003092       0.003677
##
## 2. Quantiles for each variable:
##
##     2.5%      25%      50%      75%    97.5%
## -0.72609 -0.41413 -0.20002  0.02216  0.44733
```

Heart rate slope by egg mass slope

```
corbiv <- heartratemassc$VCV[,"at.level(trait, 1):eggtempz:at.level(trait, 2)
:incday.eggID"]/
  sqrt(heartratemassc$VCV[,"at.level(trait, 1):eggtempz:at.level(trait, 1):eg
gtempz.eggID"]*
         heartratemassc$VCV[,"at.level(trait, 2):incday:at.level(trait, 2):in
```

Journal of Experimental Biology • Supplementary information

```
cday.eggID"])
summary(corbiv)

##
## Iterations = 1001:99991
## Thinning interval = 10
## Number of chains = 1
## Sample size per chain = 9900
##
## 1. Empirical mean and standard deviation for each variable,
##    plus standard error of the mean:
##
##           Mean             SD       Naive SE Time-series SE
##      -0.210326       0.259108       0.002604       0.002740
##
## 2. Quantiles for each variable:
##
##     2.5%      25%      50%      75%    97.5%
## -0.65996 -0.40489 -0.22566 -0.03451  0.33710
```

Duckling tarsus

Third, we estimate the relationship between heart rate reaction norms and tarsus reaction norms at the among-individual level.

```
prior2<-list(R=list(V=diag(2),nu=0.002),
             G=list(G1=list(V=diag(4), nu=0.002)))

heartratetarsusc<-MCMCglmm(cbind(heartratez,tarsusz)~ trait + at.level(trait,
1):eggtempz+ at.level(trait,1):incday + at.level(trait,2):ducklingage,
                  random=~us(trait+at.level(trait,1):eggtempz+at.level(trait
,2):ducklingage):eggID,
                  rcov=~us(trait):units,
                  family=c("gaussian","gaussian"),
                  nitt=100000,thin=10,burnin=1000,prior=prior2,data=datsubse
tcooling,verbose=FALSE)
```

We calculate the estimates as correlations:

Heart rate intercept by tarsus intercept

```
corR2 <- heartratetarsusc$VCV[,"traitheartratez:traittarsusz.eggID"]/
  sqrt(heartratetarsusc$VCV[,"traitheartratez:traitheartratez.eggID"]*
       heartratetarsusc$VCV[,"traittarsusz:traittarsusz.eggID"])
summary(corR2)

##
## Iterations = 1001:99991
## Thinning interval = 10
## Number of chains = 1
## Sample size per chain = 9900
##
## 1. Empirical mean and standard deviation for each variable,
##    plus standard error of the mean:
##
##           Mean             SD       Naive SE Time-series SE
##      -0.264850       0.382016       0.003839       0.016643
##
## 2. Quantiles for each variable:
##
##      2.5%       25%       50%       75%     97.5%
## -0.878810 -0.569125 -0.297099  0.007674  0.527882
```

Heart rate intercept by tarsus slope

```
corR2 <- heartratetarsusc$VCV[,"traitheartratez:at.level(trait, 2):ducklingag
e.eggID"]/
  sqrt(heartratetarsusc$VCV[,"traitheartratez:traitheartratez.eggID"]*
       heartratetarsusc$VCV[,"at.level(trait, 2):ducklingage:at.level(trait
, 2):ducklingage.eggID"])
summary(corR2)
```

Journal of Experimental Biology • Supplementary information

```
##
## Iterations = 1001:99991
## Thinning interval = 10
## Number of chains = 1
## Sample size per chain = 9900
##
## 1. Empirical mean and standard deviation for each variable,
##    plus standard error of the mean:
##
##           Mean             SD       Naive SE Time-series SE
##      -0.120175       0.329936       0.003316       0.004966
##
## 2. Quantiles for each variable:
##
##    2.5%     25%     50%     75%   97.5%
## -0.6944 -0.3730 -0.1375  0.1167  0.5350
```

Heart rate slope by tarsus intercept

```
corbiv <- heartratetarsusc$VCV[,"at.level(trait, 1):eggtempz:traittarsusz.egg
ID"]/
  sqrt(heartratetarsusc$VCV[,"at.level(trait, 1):eggtempz:at.level(trait, 1):
eggtempz.eggID"]*
         heartratetarsusc$VCV[,"traittarsusz:traittarsusz.eggID"])
summary(corbiv)
```

```
##
## Iterations = 1001:99991
## Thinning interval = 10
## Number of chains = 1
## Sample size per chain = 9900
##
## 1. Empirical mean and standard deviation for each variable,
##    plus standard error of the mean:
##
##           Mean             SD       Naive SE Time-series SE
##       0.503443       0.358049       0.003599       0.017759
##
## 2. Quantiles for each variable:
##
##    2.5%     25%     50%     75%   97.5%
## -0.3988  0.3090  0.6011  0.7801  0.9331
```

Heart rate slope by tarsus slope

```
corbiv <- heartratetarsusc$VCV[,"at.level(trait, 1):eggtempz:at.level(trait,
2):ducklingage.eggID"]/
  sqrt(heartratetarsusc$VCV[,"at.level(trait, 1):eggtempz:at.level(trait, 1):
eggtempz.eggID"]*
         heartratetarsusc$VCV[,"at.level(trait, 2):ducklingage:at.level(trait
```

Journal of Experimental Biology • Supplementary information

```
, 2):ducklingage.eggID"])
summary(corbiv)

##
## Iterations = 1001:99991
## Thinning interval = 10
## Number of chains = 1
## Sample size per chain = 9900
##
## 1. Empirical mean and standard deviation for each variable,
##    plus standard error of the mean:
##
##           Mean             SD       Naive SE Time-series SE
##      -0.186272       0.327109       0.003288       0.006584
##
## 2. Quantiles for each variable:
##
##     2.5%      25%      50%      75%    97.5%
## -0.73398 -0.43801 -0.21637  0.04407  0.49149
```

Table S1. Raw data.

Available for download at
https://journals.biologists.com/jeb/article-lookup/doi/10.1242/jeb.247120#supplementary-data
